# Lipid metabolism and osteonecrosis: unraveling causal mechanisms via multi-omics and mendelian randomization

**DOI:** 10.3389/fphys.2025.1642153

**Published:** 2025-10-23

**Authors:** Ying Chen, Zihong Zhou, Hui Che, Ding Li

**Affiliations:** ^1^ Department of Orthopedics, The Affiliated Wuxi People’s Hospital of Nanjing Medical University, Wuxi, China; ^2^ Department of Orthopaedics, Suzhou Municipal Hospital, The Affiliated Suzhou Hospital of Nanjing Medical University, Suzhou, Jiangsu, China; ^3^ Gusu School of Nanjing Medical University, Suzhou, Jiangsu, China

**Keywords:** mendelian randomization, osteonecrosis, lipidomes, phosphatidylcholine, bidirectional causality, therapeutic targets

## Abstract

Osteonecrosis (ON), a debilitating condition marked by ischemic bone death, has been clinically linked to dysregulated lipid metabolism, yet the causal relationships and underlying genetic mechanisms remain poorly defined. This study employed bidirectional Mendelian randomization (MR) analyses using genome-wide association study (GWAS) datasets to investigate causal effects between 179 plasma lipid species and ON. Functional enrichment and protein-protein interaction (PPI) analyses were conducted to identify key genes, followed by transcriptomic validation using public datasets and experimental confirmation through qRT-PCR and immunohistochemistry. MR analysis revealed that four lipid species had protective effects against ON, while two were associated with increased risk. Conversely, ON itself was found to induce a significant “lipid storm,” elevating 25 circulating lipid species, including phosphatidylcholines and triacylglycerols. PPI network analysis identified key regulatory hubs, with transcriptomic and experimental validation confirming significant dysregulation of APOE, PRKCA and ALK in osteonecrotic cartilage. These findings establish a bidirectional causal link between lipid dysregulation and ON and highlight novel molecular targets that may inform future therapeutic strategies.

## 1 Introduction

Osteonecrosis (ON) is a debilitating skeletal disorder characterized by ischemic bone cell death and structural collapse, arising from multifactorial etiologies, including vascular injury, mechanical stress, and metabolic dysregulation (Konarski et al., 2022; Keating, 2006; Glueck et al., 2008). While traumatic events are well-recognized contributors to ON pathogenesis, nontraumatic cases often idiopathic remain poorly understood, implicating complex interactions between genetic susceptibility, lipid metabolism dysregulation, and coagulation abnormalities (Hines et al., 2021; Chang et al., 2020; Seamon et al., 2012; Sekiya et al., 2010). Notably, dyslipidemia, defined by elevated triglycerides (TG), increased low-density lipoprotein cholesterol (LDL-C), and reduced high-density lipoprotein cholesterol (HDL-C), has been consistently associated with ON progression. Clinical evidence indicates that aberrant lipid profiles are prevalent in idiopathic, steroid-induced, and alcohol-associated ON, suggesting that lipid accumulation may compromise intraosseous blood flow, thereby exacerbating ischemia and necrosis (Santos et al., 2016; Borén et al., 2020; Motta et al., 2022). However, whether lipid abnormalities are causal drivers of ON or merely secondary consequences remains unresolved, underscoring the need for causal inference methodologies to disentangle these complex relationships (Ottensmann et al., 2023; Fahy et al., 2009).

Advances in lipidomics have revolutionized our understanding of lipid diversity, enabling precise quantification of lipid species such as phosphatidylcholines (PCs), triacylglycerols (TAGs), and cholesterol esters (CEs) (Hornburg et al., 2023; Levy et al., 2001). These lipids regulate critical biological processes, including redox homeostasis, inflammatory signaling, and energy metabolism, with dysregulation implicated in cardiometabolic diseases, neurodegeneration, and aging (Serhan, 2014; van Meer et al., 2008; Yap et al., 2023; Eichelmann et al., 2022). Emerging evidence suggests specific lipid subspecies, rather than broad lipid classes, may serve as biomarkers or mediators of ON (Rahman et al., 2023; Wang et al., 2021a). Yet, existing studies predominantly focus on conventional lipid panels (e.g., HDL-C, LDL-C), leaving the role of lipidomic complexity in ON largely unexplored.

Mendelian randomization (MR), a genetic instrumental variable approach, offers a robust framework to infer causality by leveraging genetic variants as proxies for modifiable exposures (Yu et al., 2024; Yu et al., 2023). Unlike observational studies prone to confounding and reverse causation, MR minimizes these biases through the random allocation of alleles during gamete formation (Yan et al., 2022; Kabata et al., 2005). While prior MR studies have linked lipid traits to musculoskeletal disorders (Weinstein et al., 2000; Motta et al., 2022), no study has systematically evaluated the bidirectional causal relationships between detailed lipidomic profiles and ON. Furthermore, the molecular mechanisms underlying lipid-ON interactions, as well as the target genes involved (e.g., oxidative stress, osteoblast dysfunction, or vascular impairment), remain speculative, necessitating integrative multiomics investigations.

In this study, we employ bidirectional two-sample MR to assess causal links between 179 lipid species and ON risk using large scale genome wide association study (GWAS) data. We further integrate transcriptomic profiling of osteonecrotic cartilage to validate candidate genes and pathways. Our findings reveal bidirectional lipid-ON associations, identify protective and pathogenic lipid subspecies, and uncover key regulatory networks such as APOE. This work advances our understanding of lipid-driven ON pathogenesis and provides actionable targets for therapeutic intervention.

## 2 Materials and methods

### 2.1 Data sources

The dataset on 179 lipid species used in this study were obtained from the GWAS Catalog database. This study involved a genetic analysis of the plasma lipidomes in 7,174 participants, focusing on 179 lipid species. The strengths of this study are evident. It features a large sample size derived from the European population and utilizes highly precise lipidomic measurements, ensuring the reliability of the data. All data can be accessed through the website https://www.ebi.ac.uk/gwas/home, with GWAS IDs ranging from GCST90277238 to GCST90277416 (Ottensmann et al., 2023).

The database for ON comes from the FinnGen study (r10. finngen. Fi) and includes 297 cases with 411,884 controls. The FinnGen study is a large-scale genomics program that analyzes more than 500,000 Finnish biobank samples and links genetic variants to health data to understand disease mechanisms and predisposition. The project is carried out by research institutions and biobanks within Finland in cooperation with international industry partners. We would like to express our gratitude to the participants and investigators of the Finnish Genetics Study (Kurki et al., 2023). Each participating cohort had previously obtained ethical approvals, so no additional ethical approvals or informed consents were required. The data utilized in this study were sourced exclusively from European population samples, which were drawn from independent GWAS databases. This approach was chosen to minimize overlap and reduce potential bias.

Additionally, this study incorporated transcriptomic analysis using publicly available gene expression data from the Gene Expression Omnibus (GEO) database. We analyzed the dataset GSE74089, which contains gene expression profiles of hip articular cartilage from patients with necrosis of the femoral head (NFH). Additionally, GSE123568 includes peripheral serum transcriptomic data derived from 10 healthy individuals and 30 patients with steroid-induced osteonecrosis of the femoral head (SONFH) at different stages of disease progression (Lu et al., 2024).

### 2.2 Selection of instrumental variables and MR discovery statistical analysis

This study followed the STROBE-MR guideline and the key principles of the Strengthening the Reporting of Observational Studies in Epidemiology (STROBE) guidelines (Elm et al., 2007) (Supplementary Table S1).

We identified SNPs associated with 179 lipid traits using a genome-wide significance threshold of p < 5 × 10^−6^, applying the same threshold for ON as the exposure (Cao et al., 2024). To minimize linkage disequilibrium (LD) bias, SNPs within a 10,000 kb window and *R* ^2^ > 0.001 were excluded. To address weak instrument bias, we calculated the F-statistic, including only SNPs with F > 10 in the MR analysis (Supplementary Tables S2 and S3) (Guan et al., 2023; Pierce et al., 2011).

For causal inference, we conducted two-sample MR using the inverse variance weighted (IVW) method as the primary approach, given its precision under valid instrumental variables (IVs). To account for potential violations of IV assumptions, we included MR-Egger regression, along with Weighted Median, Weighted Mode, and Simple Mode analyses for robustness (Burgess and Thompson, 2017; Burgess et al., 2023; Bowden et al., 2016).

Sensitivity analyses included Cochran’s Q test and Rucker’s Q test to assess heterogeneity and MR-Egger regression to detect horizontal pleiotropy (Rücker et al., 2011; Bowden et al., 2017). We performed leave-one-out analyses to evaluate individual SNP influence and used funnel plots to check for potential IVs bias (Hemani et al., 2018a; Hemani et al., 2018b; Tang and Liu, 2000).

### 2.3 Functional enrichment and network analysis

Following the identification of instrumental variables via MR, we extracted the nearest gene for each selected SNP using the FinnGen study (Supplementary Table S6). To explore the biological relevance of these genes, we performed Gene Ontology (GO) and Kyoto Encyclopedia of Genes and Genomes (KEGG) pathway enrichment analyses using the Database for Annotation, Visualization, and Integrated Discovery (DAVID) Bioinformatics Resources. These analyses provided insights into the functional enrichment and biological pathways associated with the identified genes.

To further investigate the interactions among these genes, we utilized the Search Tool for the Retrieval of Interacting Genes/Proteins (STRING) database to construct a protein-protein interaction (PPI) network. The network was then analyzed using cytoscape, where we applied the betweenness centrality method to identify potential hub genes. This approach enabled us to pinpoint key regulatory genes that may play a crucial role in the pathophysiology of osteonecrosis.

### 2.4 Transcriptomic validation using GEO dataset

To further validate the association between these key genes and osteonecrosis (ON), we incorporated transcriptomic data from publicly available datasets in the GEO database. Initial data preprocessing was performed, including gene annotation based on platform specifications and annotation files, to ensure consistency and interpretability. We then extracted the mRNA expression levels of the top 10 hub genes identified from the PPI-Hub network analysis and conducted t-tests to assess whether their expression differed significantly in each of the two datasets. The intersection of significantly differentially expressed genes across both datasets was considered as the final validation criterion. This analysis provides supporting evidence for the findings derived from our MR and PPI-Hub analyses.

### 2.5 Clinical sample collection

The normal hip articular cartilage was collected from the proximal femur during routine hip replacement (4 male and 2 female, average 72.4), while the cartilage of the NFH came from the long-term complications of patients with femoral head fractures (3 male and 3 female, average 68.8) at the Affiliated Suzhou Hospital of Nanjing Medical University according to institutional approval from the hospital ethics committee (K-2024-179-K01) and with written informed donor consent.

### 2.6 Immumohistochemical staining (IHC)

IHC was performed as in our previous study (Che et al., 2020). The primary antibodies were used as followed: SCD (23393-1-AP, Proteintech, at dilution of 1:200), APOE (66830-1-Ig, Proteintech, at dilution of 1:200), CYP7A1 (18054-1-AP, Proteintech, at dilution of 1:200), and FADS1 (10627-1-AP, Proteintech, at dilution of 1:200). The secondary antibodies were biotinylated goat anti-mouse or anti-rabbit IgG (Sigma, at dilution of 1:800). The positive cells rate is the ratio of the number of positive nuclei or/and cytoplasm to the number of all hematoxylin-labeled cells. IHC was performed using standard protocols. Due to tissue heterogeneity and morphological differences, quantitative comparison focused on calculating the proportion of positively stained cells using ImageJ-assisted thresholding. We acknowledge that single-cell level quantification (e.g., signal intensity per cell area) using higher magnification may offer greater resolution but was not performed in this study. We have now clarified this limitation.

### 2.7 RNA extraction and quantitative real-time PCR (qRT-PCR)

qRT-PCR was performed as in our previous study (Che et al., 2020). Total RNA was extracted from cartilage tissues with TRIzol reagent (R0016, Beyo-time), and RNA reverse transcribed to cDNA was used PrimeScript RT Master Mix (RR037Q, Perfect Real Time, TaKaRa). The qRT-PCR primer sequences were used as followed: ALK Forward-TCTCATCGCAGCCGATATGG, Reverse-GGCATCTCCTTAGAACGCTCT; APOE Forward-GTTGCTGGTCACATTCCTGG, Reverse-GCAGGTAATCCCAAAAGCGAC; PRKCA Forward-GTCCACAAGAGGTGCCATGAA, Reverse-AAGGTGGGGCTTCCGTAAGT; GAPDH Forward-GGAGCGAGATCCCTCCAAAAT, Reverse-GGCTGTTGTCATACTTCTCATGG, was used for normalization. Samples were processed in three independent runs to remove run-to-run variation. Relative mRNA expression was normalized to the geometric mean of the reference gene GAPDH and calculated by the 2^−ΔΔCt^ method.

### 2.8 Data analysis

All statistical analyses were conducted using the “TwoSampleMR” package in RStudio (version 4.3.1) and GraphPad Prism software (version 9), with statistical significance set at *P < 0.05, **P < 0.01, ***P < 0.001. T-tests were utilized for statistical comparisons for IHC and RT-PCR, and results were presented as mean ± standard deviation (SD).

## 3 Results

### 3.1 Investigating the causal relationship between lipidomes and ON through MR discovery analysis

**FIGURE 1:**
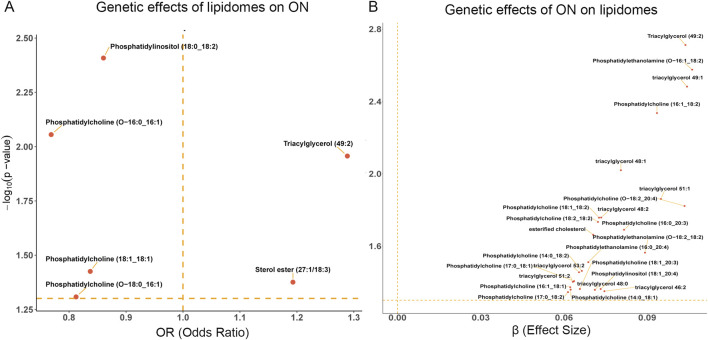
Causal effects of lipidomes and ON. **(A)** The volcano plot of the causal effect of lipidomes on ON. **(B)** The volcano plot of the causal effect of ON on lipidomes.

We observed a significant positive correlation between the levels of two specific plasma lipidomes (Sterol ester (27:1/18:3) and Triacylglycerol (49:2)) increased the incidence of ON. These findings suggest that elevated concentrations of these lipidomes may contribute to an increased risk of developing this condition. In contrast, higher levels of four distinct plasma lipidomes (Phosphatidylcholine (18:1_18:1), Phosphatidylcholine (O-16:0_16:1), Phosphatidylcholine (O-18:0_16:1), and Phosphatidylinositol (18:0_18:2)) were inversely associated with ON risk. This inverse relationship implies that these lipids might possess protective properties that mitigate the likelihood of ON (Figure 1A; Supplementary Table S4).

Conversely, our primary IVW-MR analysis demonstrated that ON was causally associated with 25 lipidomes, all of which exhibited a positive causal relationship, indicating that ON led to an increase in the levels of these lipidomes. These 25 lipidomes included esterified cholesterol, 11 isoforms of phosphatidylcholine (14:0_18:1, 14:0_18:2, 16:0_20:3, 16:1_18:1, 16:1_18:2, 17:0_18:1, 17:0_18:2, 18:1_18:2, 18:1_20:3, 18:2_18:2, O-18:2_20:4), 3 isoforms of phosphatidylethanolamine (16:0_20:4, O-16:1_18:2, O-18:2_18:2), Phosphatidylinositol (18:1_20:4), and 9 isoforms of Triacylglycerol (46:2, 48:0, 48:1, 48:2, 49:1, 49:2, 51:1, 51:2, 53:2) (Figure 1B; Supplementary Table S5). These findings suggest that ON may trigger a “lipid storm”, leading to a widespread elevation of multiple lipid species. This lipid dysregulation may reflect ON-associated metabolic disturbances and potentially contribute to disease progression and severity.

### 3.2 Robustness assessment and sensitivity analysis confirm the reliability of MR discovery findings

**FIGURE 2:**
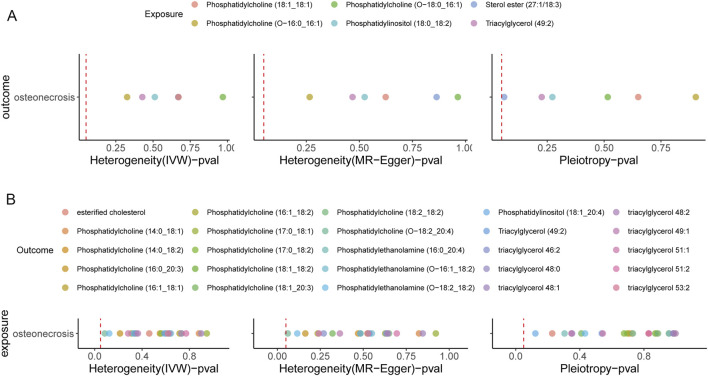
Sensitivity analysis of causal relationships. **(A)** Sensitivity analysis results for the causal effect of lipidomes on ON. **(B)** Sensitivity analysis results for the causal effect of ON on lipidomes. The red dashed line represents a p-value of 0.05, serving as a threshold for statistical significance.

To assess the robustness of our findings, we conducted multiple sensitivity analyses. Cochran’s Q test, which is used in the IVW method, and Rucker’s Q test, which is applied in the MR-Egger method, both confirmed the absence of heterogeneity (p > 0.05). Additionally, MR-Egger regression showed no evidence of pleiotropy (p < 0.05), further supporting the validity of our results (Figure 2). Leave-one-out analyses revealed no single SNP with a significant impact on MR results (Supplementary Figures S1, S3). And funnel plot analyses provided additional support for the reliability and consistency of our findings (Supplementary Figures S2, S4).

### 3.3 Functional enrichment and hubs gene network analysis identify key Lipidomes-ON related genes

After identifying significant lipidomes associated with ON through bidirectional MR analysis, we further investigated the underlying biological mechanisms by mapping the differentially associated SNPs of exposure variables to their corresponding genes (Supplementary Table S6).

**FIGURE 3:**
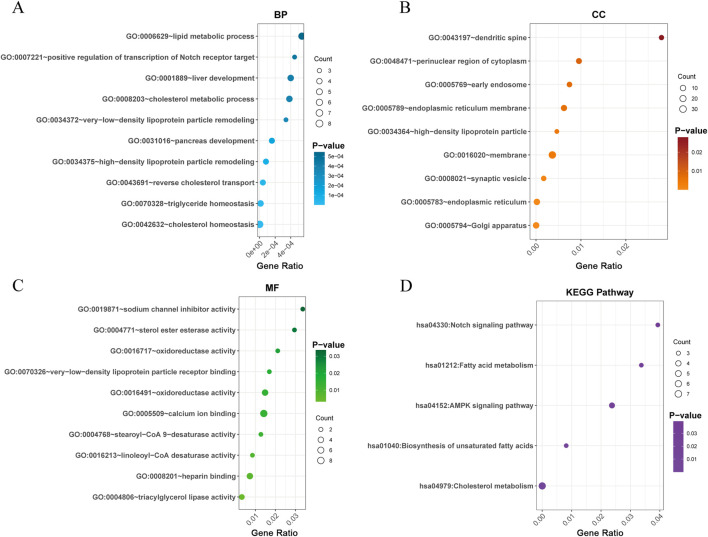
Functional enrichment analysis of lipidomes-ON-related genes. **(A)** GO analysis of biological processes enriched in lipidomes-ON-related genes. **(B)** GO analysis of cellular components enriched in lipidomes-ON-related genes. **(C)** GO analysis of molecular functions enriched in lipidomes-ON-related genes. **(D)** KEGG pathways enriched in lipidomes-ON-related genes.

To elucidate the biological significance of the identified genes, GO and KEGG pathway enrichment analyses were conducted. The GO analysis revealed that the genes associated with lipidomes-ON-related changes were significantly enriched in biological processes (BP) such as cholesterol homeostasis, triglyceride homeostasis and reverse cholesterol transport, proving a central role of lipid regulation in ON pathogenesis (Figure 3A). In terms of cellular components (CC), the enriched terms included Golgi apparatus, endoplasmic reticulum, and synaptic vesicle (Figure 3B). The molecular function (MF) enrichment indicated significant involvement in lipid receptor binding, sterol ester esterase activity, and oxidoreductase activity, reinforcing their roles in lipid metabolism and oxidative stress regulation (Figure 3C).

The KEGG pathway analysis identified several key pathways involved in lipidomes-ON related pathogenesis, including the Notch signaling pathway, fatty acid (FA) metabolism, AMPK signaling pathway, biosynthesis of unsaturated FA and cholesterol metabolism (Figure 3D). These pathways play crucial roles in maintaining lipid homeostasis, energy metabolism, and cellular differentiation, all of which are closely linked to bone health and osteonecrosis progression.

**FIGURE 4:**
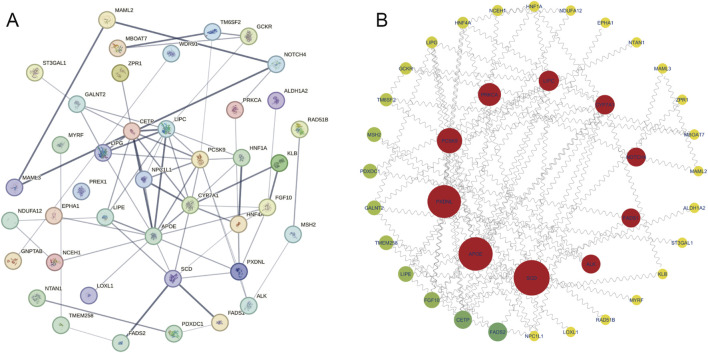
PPI network analysis. **(A)** PPI network of lipidomes-ON-related genes. **(B)** Top regulatory hub genes in the PPI network.

To further explore the interactions between lipidomes-ON-related genes, PPI network was constructed (Figure 4A). The network analysis revealed multiple interconnected clusters, suggesting a coordinated regulatory network among lipidomes-ON-related genes. To identify hub genes in the PPI network, we applied the Degree algorithm in the cytoHubba plugin of Cytoscape, which ranks nodes based on their connectivity (node degree) and network centrality. The top 10 genes with the highest node degrees were selected as hub genes. As a result, SCD, APOE, PXDNL, PCSK9, PRKCA, LIPC, CYP7A1, NOTCH4, FADS1, and ALK were identified as core regulatory genes within the network (Figure 4B).

### 3.4 Validation of lipidomes-ON-related genes in ON through transcriptomic analysis

**FIGURE 5:**
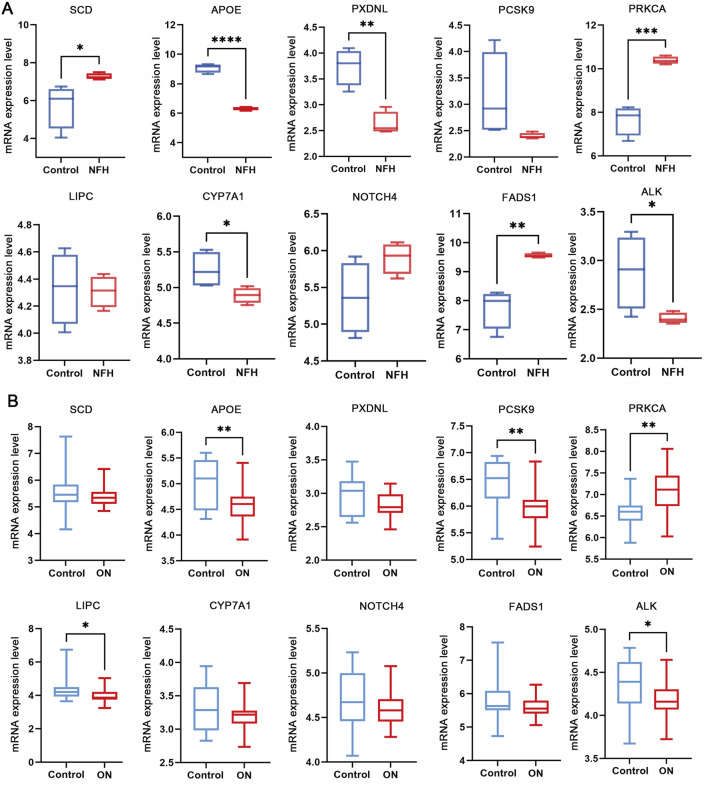
Differential mRNA expression analysis of lipidomes-ON-related gene using **(A)** the GSE74089 and **(B)** GSE123568 transcriptomic dataset. (NFH, necrosis of the femoral head; ON, Osteonecrosis).

To further validate the association between predicted lipidomes-ON-related Genes and ON, we conducted transcriptomic analysis using the GSE74089 and GSE123568 dataset. Differential expression analysis was performed to assess whether the top 10 genes identified from discovery analysis were significantly altered at the transcriptional level in ON. In the GSE74089 dataset, several genes exhibited significant differential expression between ON samples and controls. Specifically, SCD, FADS1, and PRKCA were significantly upregulated in ON cartilage compared to controls, whereas APOE, PXDNL, ALK and CYP7A1 were markedly downregulated (Figure 5A). In the GSE123568 dataset, serum transcriptomic analysis revealed that APOE, PCSK9, LIPC and ALK were significantly downregulated in the disease group, while PRKCA was significantly upregulated (Figure 5B). Notably, APOE, PRKCA and ALK showed consistent expression patterns across both datasets. These findings provide independent transcriptomic evidence supporting our MR-derived causal associations, strengthening the hypothesis that lipid metabolism dysregulation plays a crucial role in ON.

### 3.5 Validation of lipidomes-on-related genes in ON through experimental analysis

**FIGURE 6:**
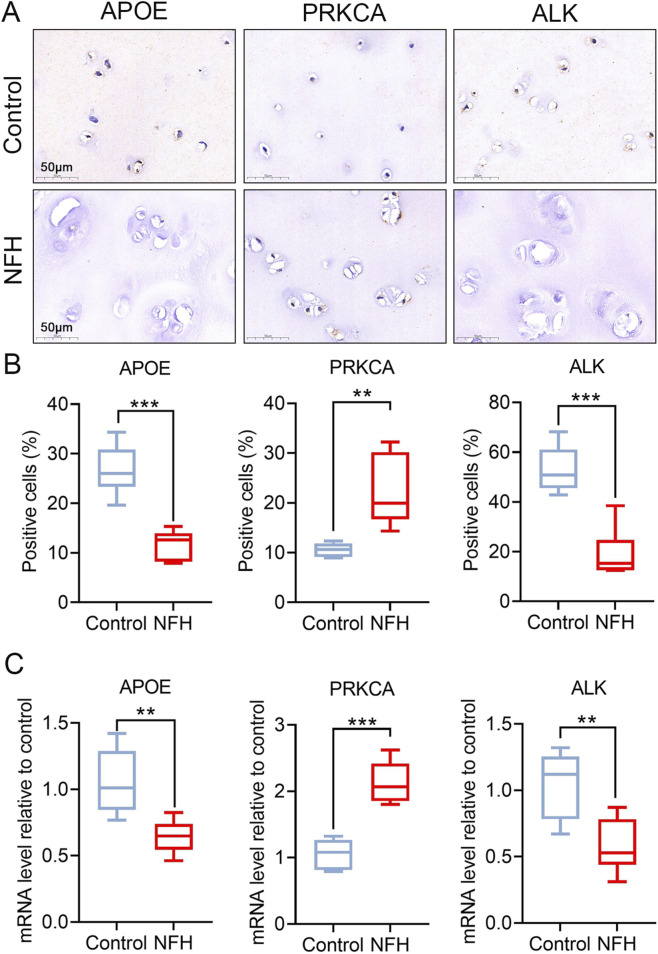
Experimental validation of lipidomes-ON-related genes. **(A)** Representative micrographs of slices stained immunohistochemically for SCD, PRKCA and ALK. **(B)** Percentage of total immuno-positive cells (%). **(C)** Target mRNA expression as assessed by qRT-PCR relative to GAPDH expression. Data are presented with mean ± SD (n = 6); *p < 0.05, **p < 0.01, ***p < 0.001.

IHC and qRT-PCR analyses were conducted using normal and osteonecrotic cartilage from the hip joint to further verify the accuracy of the predicted lipidomes-ON-related genes at the protein and mRNA levels. As a result, APOE, PRKCA and ALK exhibited significant differences in expression. PRKCA showed upregulated protein levels in ON compared to control samples, while APOE and ALK exhibited decreased levels (Figures 6A,B). This trend was consistent with the qRT-PCR (Figure 6C) and transcriptomic evidence above. These findings proved these four genes may play an undeniable role in the lipid metabolism dysregulation and contribute to ON development.

## 4 Discussion

ON, a devastating condition marked by ischemic bone death, remains etiologically enigmatic despite associations with vascular disruption, mechanical stress, and metabolic dysfunction (Lespasio et al., 2019; Mankin, 1992; Chang et al., 1993). A major strength of our study lies in its integrative multi-omics framework, which combines bidirectional MR analysis, transcriptomic profiling, and experimental validation. This systems-level approach allows us to triangulate evidence across genetic, transcript, and protein levels, thereby enhancing the robustness and biological relevance of our findings. By leveraging multiple independent data modalities, we provide a comprehensive and multidimensional view of the lipid-osteonecrosis axis that would not be achievable through a single method alone.

The relationship between lipidomes and ON is significant. Elevated levels of blood lipids, such as cholesterol and triglycerides, are linked to the development of ON. Abnormal lipid metabolism can lead to the formation of fat emboli, which obstruct blood vessels and reduce blood flow to the bone, causing ischemia and subsequent bone cell death (Yu et al., 2024; Yu et al., 2023). Studies have demonstrated that lipid abnormalities are prevalent in patients with nontraumatic ON, indicating a direct link between dyslipidemia and the disease (Wang et al., 2021b). Research has shown that managing lipid levels through lifestyle changes and medications can help prevent or slow the progression of ON. For instance, changes in bone tissue lipids are significant in individuals with steroid- and alcohol-induced ON (Brett et al., 2021). Another study emphasized the role of abnormal lipid profiles in the development of nontraumatic ON, highlighting the importance of monitoring and managing blood lipid levels to mitigate the risk (Yan et al., 2022).

We identified phosphatidylcholines (PCs) as protective lipid species, with PC (18:1_18:1), PC (O-16:0_16:1), and PC (O-18:0_16:1) inversely associated with ON risk. Some studies have reported that PCs are critical for membrane integrity, lipid transport, and anti-inflammatory signaling (Kent, 2005; Gibellini and Smith, 2010; Cantley, 2002). Their deficiency may compromise vascular endothelial function or osteoblast survival, exacerbating ischemia in weight-bearing bones like the femoral head. This aligns with clinical observations of reduced PC levels in steroid-induced ON and highlights their therapeutic potential (Ren et al., 2018). Conversely, sterol ester (27:1/18:3) and triacylglycerol (TAG 49:2) exhibited pro-osteonecrotic effects. TAG (49:2), enriched in glucocorticoid-associated ON, may promote lipotoxicity via adipocyte hypertrophy or impaired β-oxidation, while sterol ester accumulation could reflect dysregulated cholesterol efflux, as suggested by SREBF1 polymorphisms in ON cohorts (Lee et al., 2009; Wang et al., 2021c). Our MR study provides exploratory insights into the potential causal interplay between lipidomic profiles and ON, suggesting bidirectional associations that highlight lipidomes as both potential contributors to and consequences of bone pathology. While we applied standard sensitivity analyses, including MR-Egger intercept, Cochran’s Q, and leave-one-out analysis to evaluate heterogeneity and pleiotropy, we acknowledge that residual horizontal pleiotropy and violation of MR assumptions cannot be fully excluded. Horizontal pleiotropy, where genetic variants influence the outcome through pathways independent of the exposure, can bias causal estimates. Although the MR-Egger intercept test showed no significant evidence of directional pleiotropy and the leave-one-out and funnel plot analyses supported the consistency of our findings, these methods have limited power. Therefore, our results should be interpreted as hypothesis-generating and exploratory, rather than conclusive proof of causality, particularly given the complexity of traits like ON.

Notably, ON causally elevated 25 lipid species. This “lipid storm”, particularly esterified cholesterol and various phospholipids, could imply a disruption in cellular lipid homeostasis triggered by ON. Phosphatidylcholine and phosphatidylethanolamine are major components of cell membranes (Posor et al., 2022), and their altered levels could impact membrane integrity and function, potentially exacerbating cellular stress and contributing to the pathophysiology of ON (Yan et al., 2022). Similarly, phosphatidylinositol is involved in signaling pathways that regulate a variety of cellular processes, including apoptosis and inflammation, both of which are relevant to the progression of ON. Moreover, the consistent increase in triacylglycerol levels suggests enhanced lipid storage or impaired lipid utilization in affected tissues. This lipid accumulation could lead to lipotoxicity, further aggravating tissue damage and contributing to the progression of ON (Baek et al., 2022; Motta et al., 2022). Increased phosphatidylethanolamines (PEs) and PCs could reflect compensatory membrane repair mechanisms, while elevated esterified cholesterol points to disrupted reverse cholesterol transport a pathway central to atherosclerosis, now implicated in ON progression (Efrat et al., 2009).

Our integrative multiomics approach revealed critical biological pathways and regulatory hubs linking lipid metabolism dysregulation to ON pathogenesis. GO enrichment analysis highlighted the central role of lipid homeostasis processes, including cholesterol and triglyceride homeostasis and reverse cholesterol transport in ON development. These findings align with observations of lipid accumulation in necrotic bone and suggest that impaired lipid clearance mechanisms, rather than mere lipid overproduction, drive intraosseous ischemia (Franceschini et al., 1991; Bao et al., 2023; Tian and Yu, 2015). The enrichment of cellular components such as the Golgi apparatus and endoplasmic reticulum underscores the importance of intracellular lipid trafficking and membrane synthesis in osteocyte survival, as disrupted vesicular transport may exacerbate lipid droplet deposition and endoplasmic reticulum stress, triggering apoptosis in weight-bearing bones like the femoral head (Zhao, 2012).

KEGG pathway analysis further implicated including Notch signaling, AMPK signaling, and FA metabolism as key drivers of ON progression. Notch signaling, a well-established regulator of osteoblast-osteoclast crosstalk, may mediate lipid-induced bone remodeling defects (Gao et al., 2023). The Notch signaling pathway plays a crucial role in skeletal development and adult bone remodeling. In osteoblastic cell lines, Notch activation inhibits cell differentiation, leading to a reduction in trabecular bone mass. In adult mice, osteocyte-specific activation of Notch signaling significantly promotes bone formation, primarily by enhancing mineralization. This effect can reverse age-related bone loss and facilitate fracture healing. In contrast, inhibition of Notch signaling, such as γ-secretase deficiency, severely impairs the terminal differentiation of osteoblasts (Liu et al., 2016; Zanotti and Canalis, 2016). Concurrently, AMPK pathway inhibition (a sensor of cellular energy status) may disrupt FA oxidation, exacerbating lipid accumulation in bone marrow adipocytes (Tabe et al., 2017). These pathways collectively shape a metabolic microenvironment that influences vascular function and osteocyte viability.

PPI Network Construction and Hub Gene Identification analysis identified SCD and APOE as top hub genes, with SCD encoding stearoyl-CoA desaturase, a rate-limiting enzyme in monounsaturated FA synthesis (Ntambi et al., 2002). In our validation results, SCD expression was significantly upregulated in ON cartilage, potentially due to its role in promoting lipid droplet formation, which may render osteocytes more susceptible to lipotoxicity. Conversely, APOE dysregulation (a key cholesterol transporter) may impair reverse cholesterol transport, mirroring its role in atherosclerosis and suggesting shared mechanisms between vascular and bone pathologies (Toribio-Fernández et al., 2024). Notably, CYP7A1 downregulated in ON cartilage—highlight compromised detoxification and angiogenesis. CYP7A1 deficiency may reduce cholesterol catabolism, synergizing with lipid-driven ischemia (Moscat et al., 2003). Besides, modifications to the FADS genes impact the activity of polyunsaturated FA desaturation, which in turn alters the lipid composition in human blood and tissue. As a component of the FADS family, FADS1 regulates FA synthesis and plays a role in lipid metabolism (Koletzko et al., 2019). These findings collectively position lipid metabolism as a dynamic regulator of bone-vascular crosstalk, where hub genes orchestrate lipid handling, inflammatory responses, and osteogenic capacity.

Our findings, integrating bidirectional MR analysis, PPI network construction and hub gene identification analysis, transcriptomic and experimental analysis, provide a mechanistic framework for the pathogenesis of ON. Different lipid subspecies exhibit dual roles, either disrupting or stabilizing osteocyte lipid homeostasis. Meanwhile, necrotic bone releases lipid mediators that dysregulate systemic lipid circulation, perpetuating a self-reinforcing cycle. Targeting these lipidomes-ON-related key regulatory hubs may interrupt this cycle, offering novel therapeutic strategies for ON.

However, our study has certain limitations. We acknowledge that the lipidomic SNP-based gene annotations and transcriptomic data were derived from independent datasets. As such, the biological pathways identified from each may not fully overlap. While our multi-omics framework is designed to identify convergent biological signals, the generalizability of specific GO or KEGG terms across datasets should be interpreted with caution. It did not investigate the effects of varying exposure periods or levels of lipidomes on ON pathogenesis. The MR analysis was limited to a European population, raising questions about its generalizability to other populations. Although our MR analysis supports potential causal associations between lipid species and ON, we acknowledge that MR is not immune to residual confounding and horizontal pleiotropy. Future studies should explore these aspects to validate and extend our findings. Furthermore, we recognize the limitations of semi-quantitative IHC assessment, particularly under low magnification, and acknowledge that cellular morphology differences between ON and controls may influence staining interpretation. More precise protein quantification methods should be considered in future studies.

**FIGURE 7:**
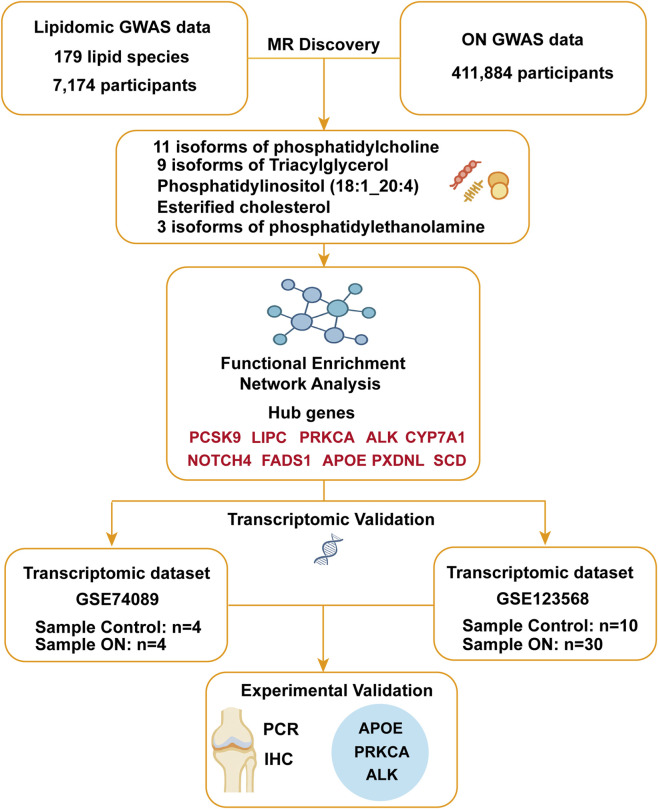
Multi-omics and Mendelian randomization study design and analytical framework.

In conclusion, this study provides the first exploratory investigation into the bidirectional associations between 179 lipid species and ON using integrated MR and multi-omics analyses. We identified lipid species that may be protective or pathogenic in the context of ON, and observed a potential ‘lipid storm’ pattern induced by ON itself. Through PPI network construction, transcriptomic, and experimental validation, several lipid metabolism–related genes, such as APOE, PRKCA and ALK, were found to be significantly dysregulated in osteonecrotic cartilage (Figure 7). While these findings offer novel insights into lipid-bone interactions and identify promising molecular candidates, they should be considered hypothesis-generating due to limitations in MR assumptions and sample size. Future studies are warranted to validate these results in independent cohorts and mechanistic models, and to explore their translational potential for therapeutic development.

## Data Availability

The original contributions presented in the study are included in the article/Supplementary Material; further inquiries can be directed to the corresponding authors.
