# Supplementary material for: Lipid metabolism and osteonecrosis: unraveling causal mechanisms via multi-omics and mendelian randomization
Source: Front Physiol. 2025 Oct 23;16:1642153. doi: 10.3389/fphys.2025.1642153 (PMC12589827; doi:10.3389/fphys.2025.1642153)
Supplement: Supplementary file 3 [file DataSheet4.pdf]

Supplementary Figure 4 Funnel plots of causal effect of osteonecrosis on lipidomes

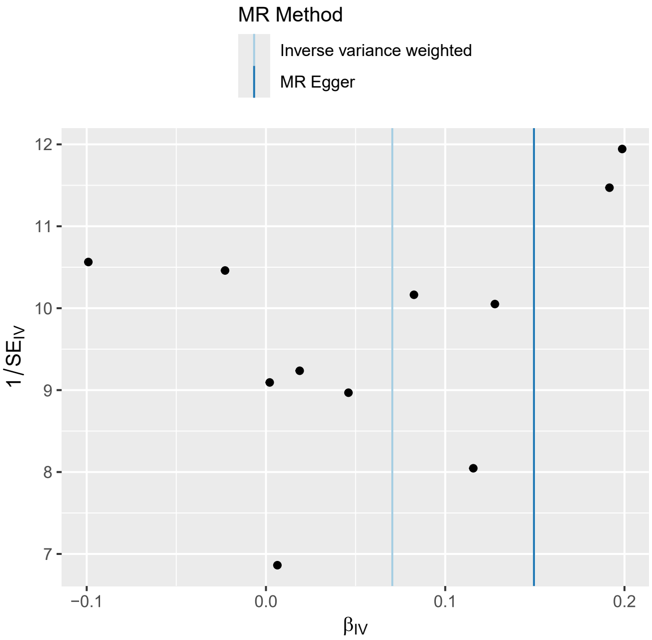

outcome: GCST90277243

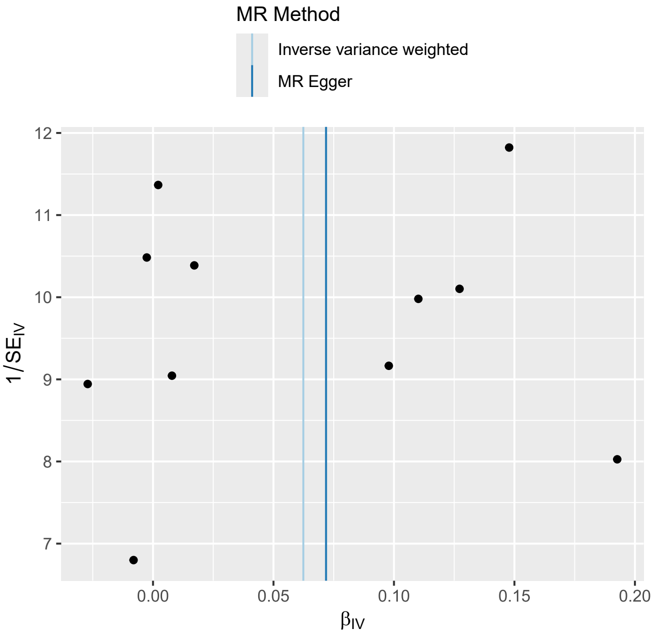

outcome: GCST90277273

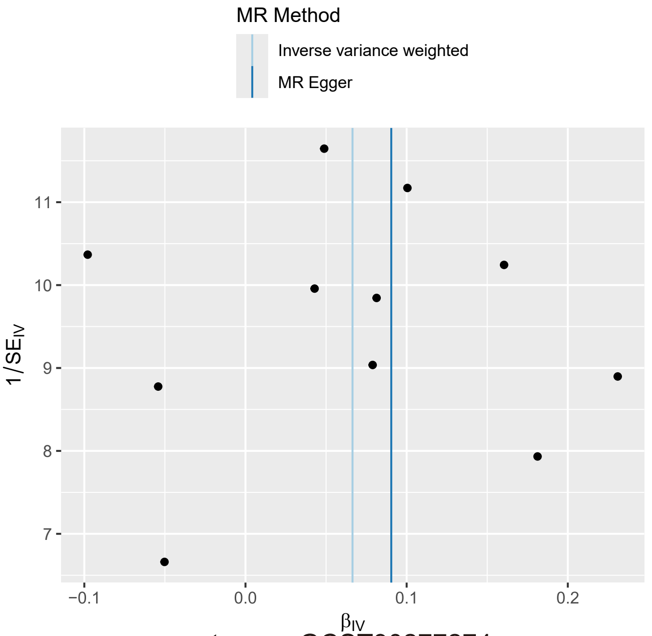

outcome: GCST90277274

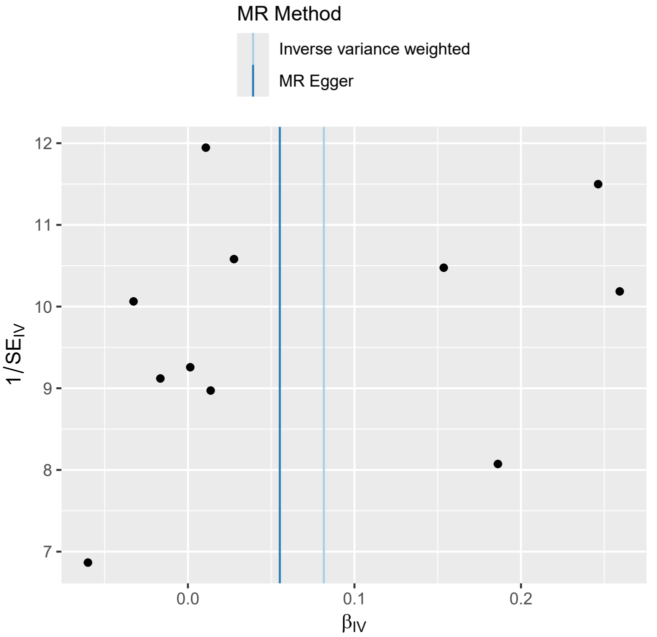

outcome: GCST90277286

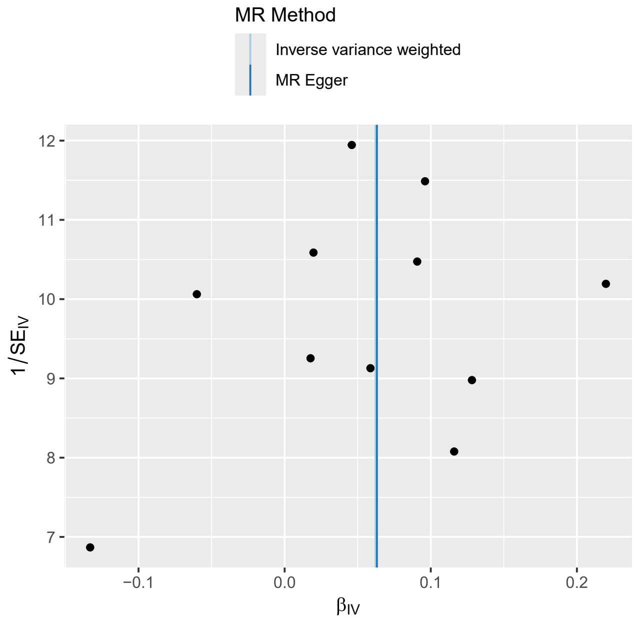

outcome: GCST90277293

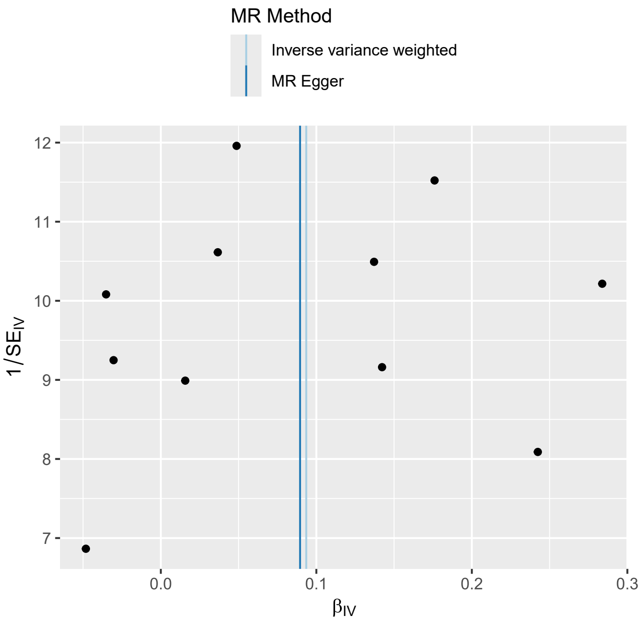

outcome: GCST90277294

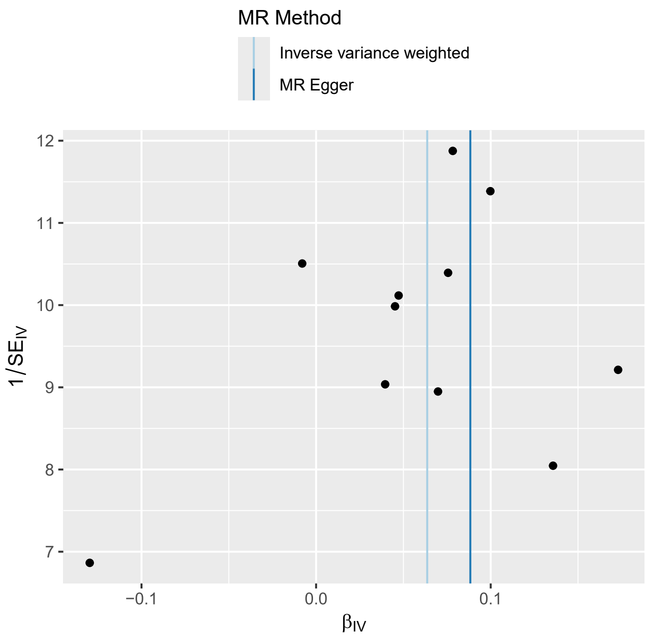

outcome: GCST90277296

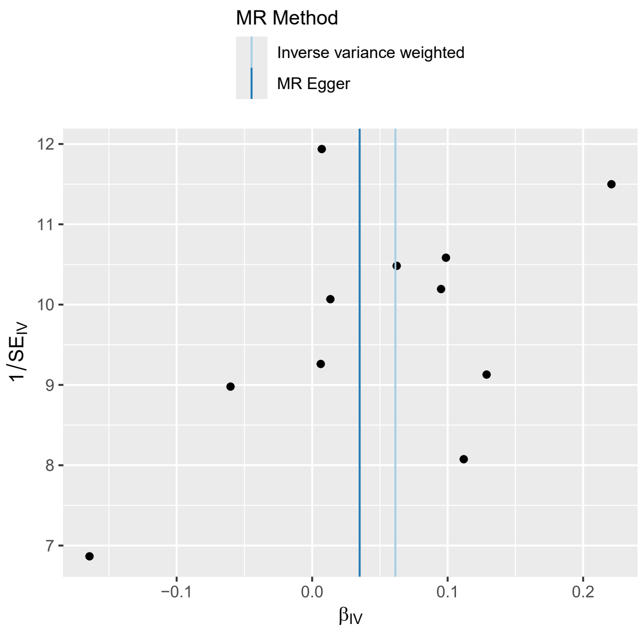

outcome: GCST90277297

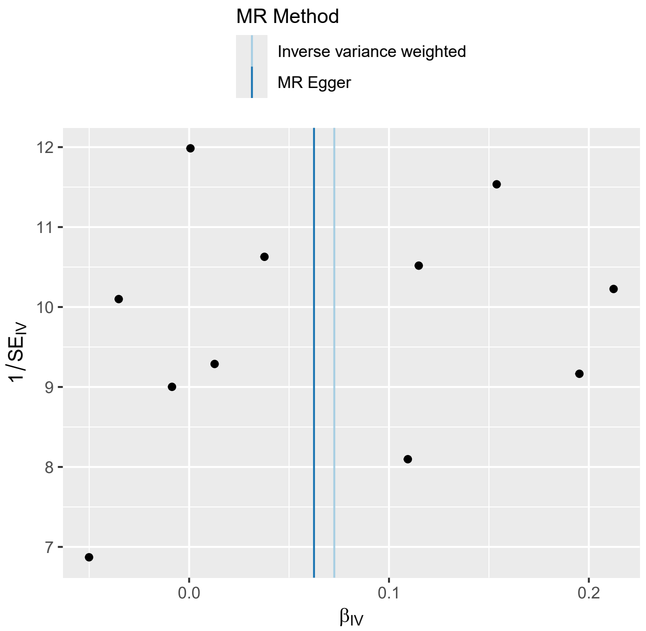

outcome: GCST90277309

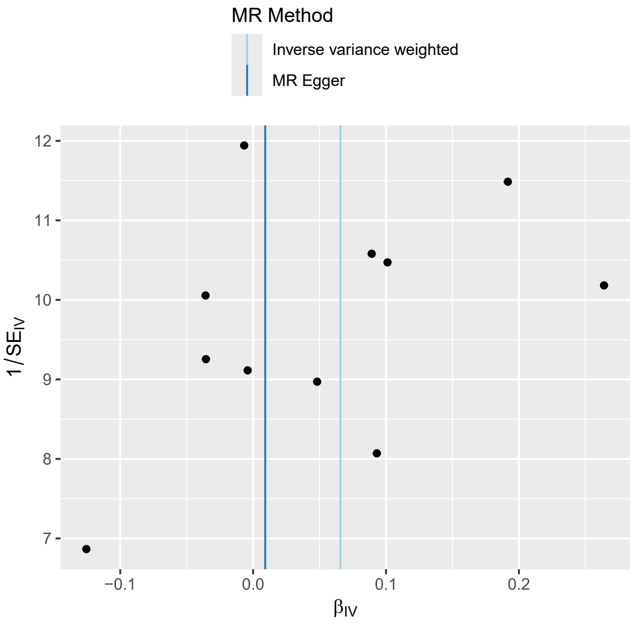

outcome: GCST90277312

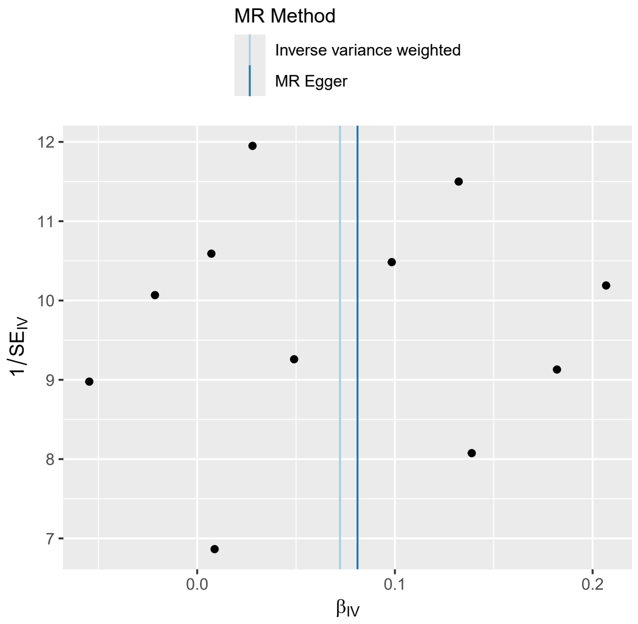

outcome: GCST90277314

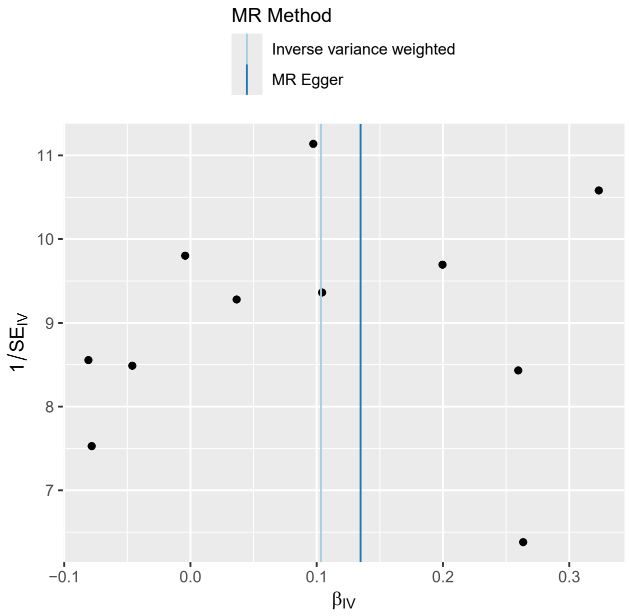

outcome: GCST90277344

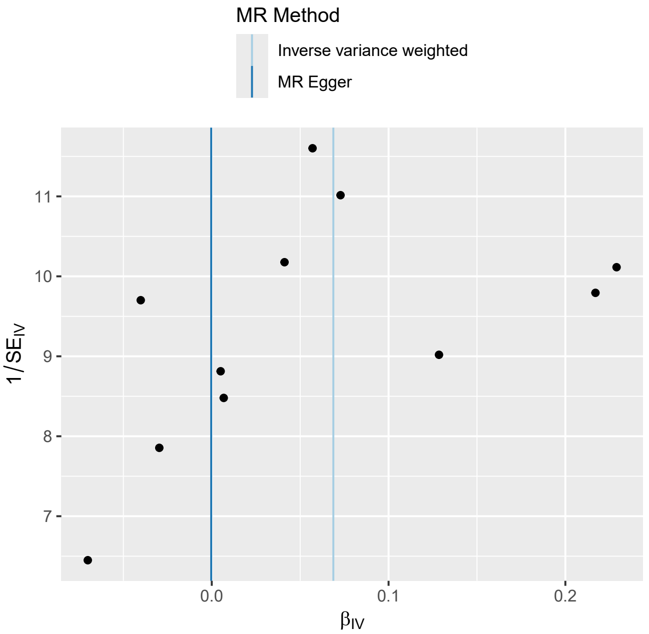

outcome: GCST90277346

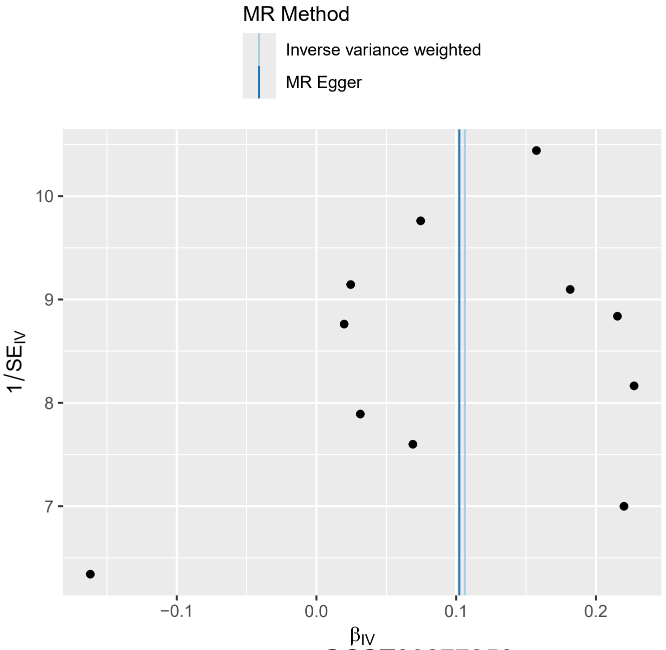

outcome: GCST90277350

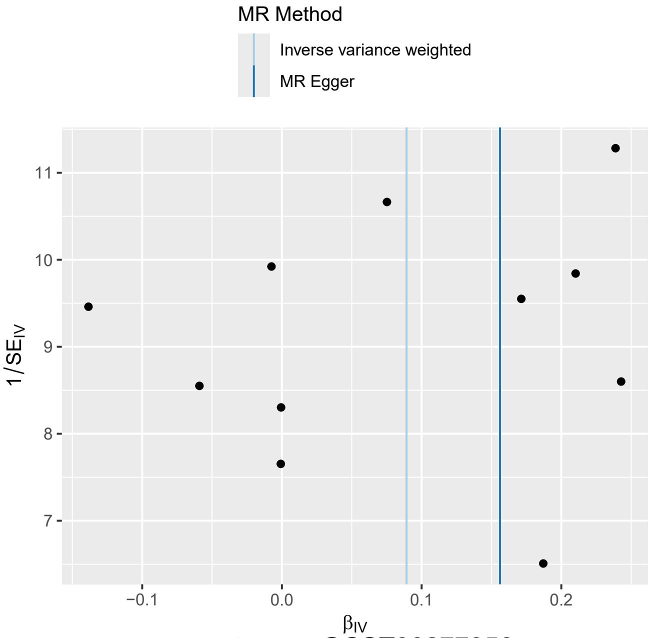

outcome: GCST90277356

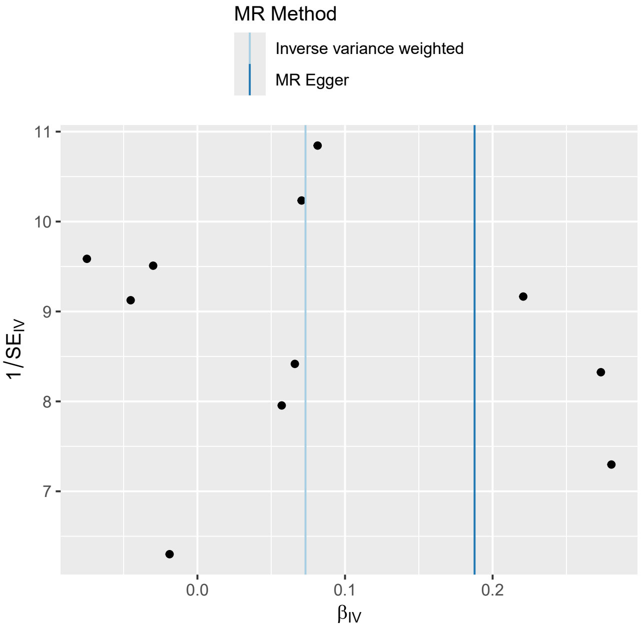

outcome: GCST90277367

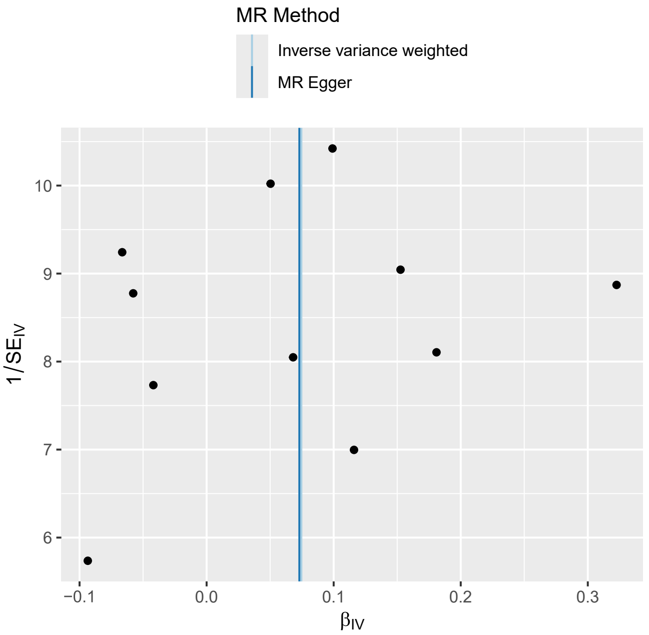

outcome: GCST90277380

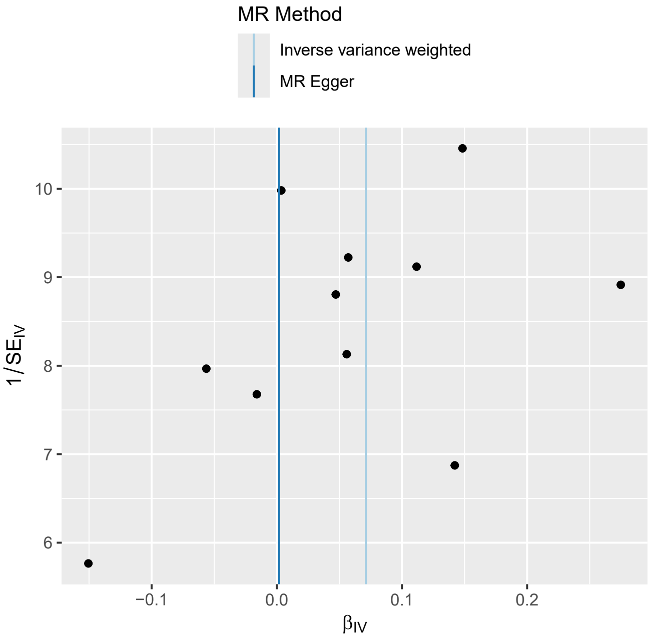

outcome: GCST90277381

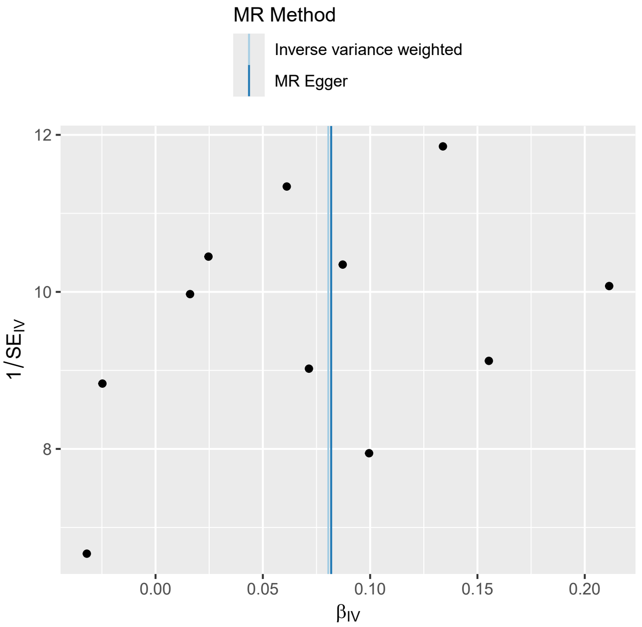

outcome: GCST90277382

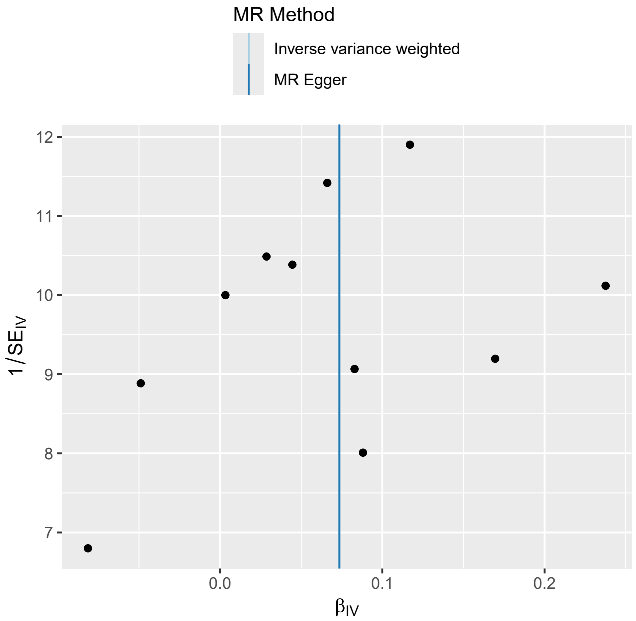

outcome: GCST90277383

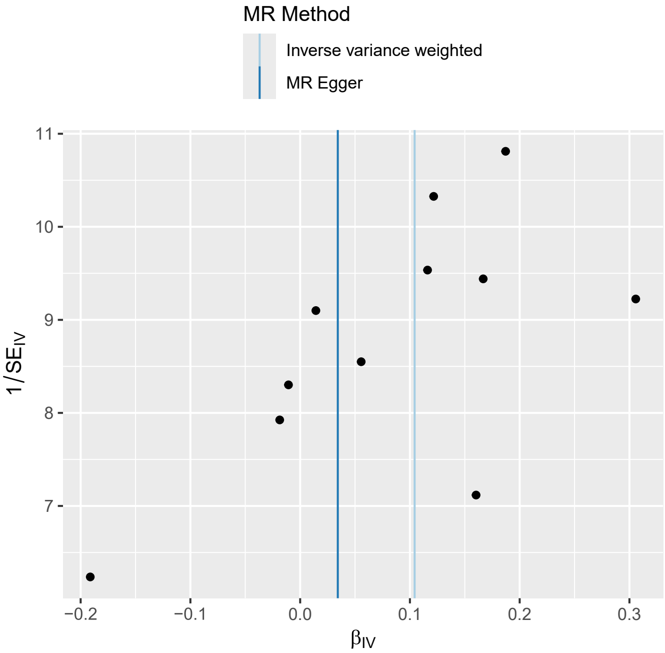

outcome: GCST90277385

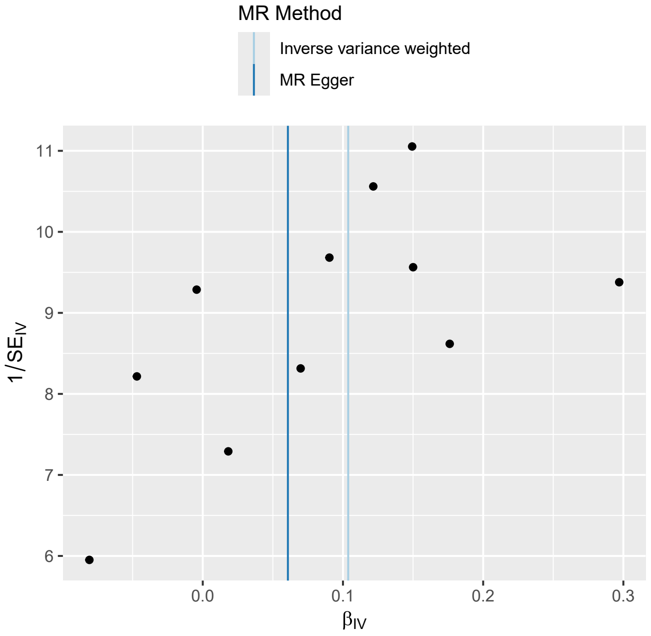

outcome: GCST90277386

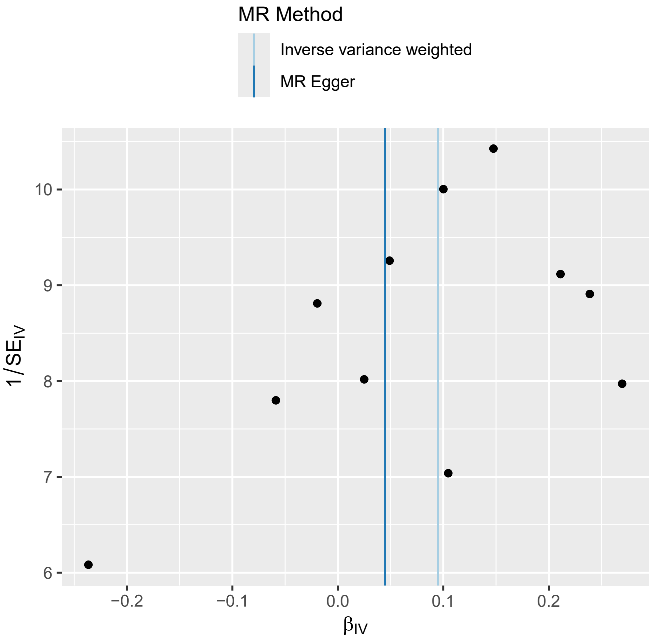

outcome: GCST90277392

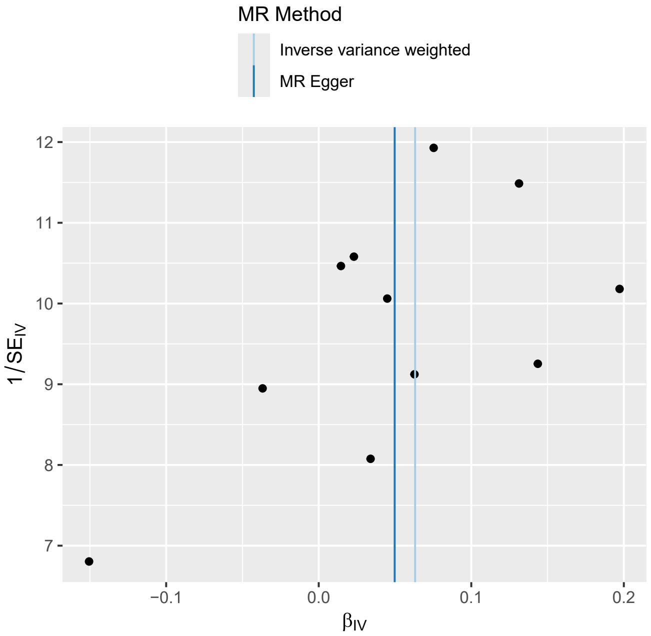

outcome: GCST90277393

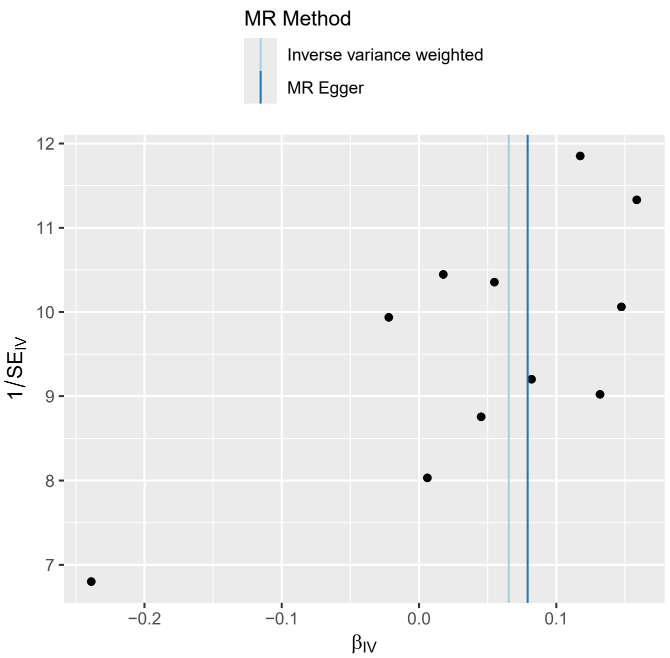

outcome: GCST90277401
