# Supplementary material for: Lipid metabolism and osteonecrosis: unraveling causal mechanisms via multi-omics and mendelian randomization
Source: Front Physiol. 2025 Oct 23;16:1642153. doi: 10.3389/fphys.2025.1642153 (PMC12589827; doi:10.3389/fphys.2025.1642153)
Supplement: Supplementary file 5 [file Table1.docx]

**STROBE-MR checklist of recommended items to address in reports of Mendelian randomization studies**^1^ ^2^

| **Item No.** | **Section** | **Checklist item** | **Relevant text from manuscript** |
| --- | --- | --- | --- |
| 1 | **TITLE and ABSTRACT** | Indicate Mendelian randomization (MR) as the study’s design in the title and/or the abstract if that is a main purpose of the study | this study utilized bidirectional Mendelian Randomization (MR) analyses to explore this causal association. |
|  | **INTRODUCTION** |  |  |
| 2 | **Background** | Explain the scientific background and rationale for the reported study. What is the exposure? Is a potential causal relationship between exposure and outcome plausible? Justify why MR is a helpful method to address the study question | Several studies propose that dysregulated lipid metabolism leads to increased intraosseous pressure and impaired blood flow, culminating in the necrosis of bone tissue. Nonetheless, the precise mechanisms underlying this association remain elusive. |
| 3 | **Objectives** | State specific objectives clearly, including pre-specified causal hypotheses (if any). State that MR is a method that, under specific assumptions, intends to estimate causal effects | our study employs a bidirectional MR method to explore the relationship between lipidomes and osteonecrosis. This approach aims to yield more definitive results, thereby offering robust evidence for clinical treatment and health guidance for patients. |
|  | **METHODS** |  |  |
| 4 | **Study design and data sources** | Present key elements of the study design early in the article. Consider including a table listing sources of data for all phases of the study. For each data source contributing to the analysis, describe the following: |  |
|  | a) | Setting: Describe the study design and the underlying population, if possible. Describe the setting, locations, and relevant dates, including periods of recruitment, exposure, follow-up, and data collection, when available. | This study utilized a bidirectional two-sample Mendelian randomization approach, examining 179 lipid species and osteonecrosis as the exposure and outcome respectively.  The dataset on 179 lipid species used in this study were obtained from the GWAS Catalog database. All data can be accessed through the website https://www.ebi.ac.uk/gwas/home, with GWAS IDs ranging from GCST90277238 to GCST90277416. The database for osteonecrosis comes from the FinnGen study (r10.finngen. Fi) and includes 297 cases with 411,884 controls. |
|  | b) | Participants: Give the eligibility criteria, and the sources and methods of selection of participants. Report the sample size, and whether any power or sample size calculations were carried out prior to the main analysis | This study involved a genetic analysis of the plasma lipidomes in 7,174 participants, focusing on 179 lipid species. The database for osteonecrosis comes from the FinnGen study (r10.finngen. Fi) and includes 297 cases with 411,884 controls. The FinnGen study is a large-scale genomics program that analyzes more than 500,000 Finnish biobank samples and links genetic variants to health data to understand disease mechanisms and predisposition. |
|  | c) | Describe measurement, quality control and selection of genetic variants | We employed a genome-wide significance threshold of p<5e-6 to identify SNPs associated with 179 lipid traits. The same threshold was applied when osteonecrosis was the exposure to ensure an adequate number of SNPs for analysis. Additionally, SNPs in linkage disequilibrium (LD) within a 10,000 kb window and an R2>0.001 with the most significant SNP were excluded to avoid LD confounding. Given the relatively lenient SNP selection threshold, we aimed to mitigate weak instrument bias by calculating the F-statistic to evaluate the strength of the instrumental variables (IVs). Only SNPs with an F-statistic greater than 10 were included in this Mendelian Randomization (MR) analysis. |
|  | d) | For each exposure, outcome, and other relevant variables, describe methods of assessment and diagnostic criteria for diseases | In the methods section, we provide the specific location where the data can be accessed. The detailed assessments and disease diagnostic criteria can be obtained via email or other contact methods. |
|  | e) | Provide details of ethics committee approval and participant informed consent, if relevant | Each participating cohort had previously obtained ethical approvals, so no additional ethical approvals or informed consents were required. |
| 5 | **Assumptions** | Explicitly state the three core IV assumptions for the main analysis (relevance, independence and exclusion restriction) as well assumptions for any additional or sensitivity analysis | The selection of these instrumental variables had to meet three key assumptions: (1) the SNPs must be strongly associated with the exposure; (2) the SNPs must not be linked to any confounding. factors. (3) the SNPs should influence the outcome solely through the exposure. |
| 6 | **Statistical methods: main analysis** | Describe statistical methods and statistics used |  |
|  | a) | Describe how quantitative variables were handled in the analyses (i.e., scale, units, model) | This research does not involve any transformations of quantitative variables |
|  | b) | Describe how genetic variants were handled in the analyses and, if applicable, how their weights were selected | For our primary method, we utilized random-effects inverse variance weighted (IVW) analysis. To further substantiate our findings and ensure the robustness of our results, we incorporated additional sensitivity analyses using Weighted Median, Weighted Mode, and Simple Mode method. In addition to our primary analyses, we conducted extensive sensitivity analyses to ensure the robustness and reliability of our positive results. Specifically, we applied Cochran's Q statistic to our MR-IVW analyses and Rucker's Q statistic to our MR-Egger analyses to determine the presence of heterogeneity among the instrumental variables. To further validate our findings, we performed a leave-one-out analysis and funnel plot analysis. |
|  | c) | Describe the MR estimator (e.g. two-stage least squares, Wald ratio) and related statistics. Detail the included covariates and, in case of two-sample MR, whether the same covariate set was used for adjustment in the two samples | we utilized random-effects inverse variance weighted (IVW) analysis, given that it is most precise when the instrumental variables (IVs) are univalent. The IVW approach is renowned for its accuracy under the assumption that all selected IVs are valid. However, in instances where the IVs exhibit multivalence, the reliability of the IVW method may be compromised. In such scenarios, MR-Egger regression emerges as a more robust alternative, as it can produce more reliable inferences even when some IVs are invalid. |
|  | d) | Explain how missing data were addressed | In this MR analysis, there is no missing data. |
|  | e) | If applicable, indicate how multiple testing was addressed | In this study, the individual exposures were independent factors, so the issue of multiple testing did not arise. |
| 7 | **Assessment of assumptions** | Describe any methods or prior knowledge used to assess the assumptions or justify their validity | Given the relatively lenient SNP selection threshold, we aimed to mitigate weak instrument bias by calculating the F-statistic to evaluate the strength of the instrumental variables (IVs). A variety of sensitivity tests were also applied to demonstrate the reliability of the results. |
| 8 | **Sensitivity analyses and additional analyses** | Describe any sensitivity analyses or additional analyses performed (e.g. comparison of effect estimates from different approaches, independent replication, bias analytic techniques, validation of instruments, simulations) | we applied Cochran's Q statistic to our MR-IVW analyses and Rucker's Q statistic to our MR-Egger analyses to determine the presence of heterogeneity among the instrumental variables. we employed the MR-Egger method to detect and account for horizontal pleiotropy, which could bias our results if unaddressed. To further substantiate our findings and ensure the robustness of our results, we incorporated additional sensitivity analyses using Weighted Median, Weighted Mode, and Simple Mode method. Also, we performed leave-one-out and funnel plot analysis. |
| 9 | **Software and pre-registration** |  |  |
|  | a) | Name statistical software and package(s), including version and settings used | All statistical analyses were conducted using the "TwoSampleMR" within RStudio (version 4.3.1). |
|  | b) | State whether the study protocol and details were pre-registered (as well as when and where) | This study was not pre-registered with the study protocol and details. |
|  | **RESULTS** |  |  |
| 10 | **Descriptive data** |  |  |
|  | a) | Report the numbers of individuals at each stage of included studies and reasons for exclusion. Consider use of a flow diagram | The dataset on 179 lipid species used in this study were obtained from the GWAS Catalog database. All data can be accessed through the website https://www.ebi.ac.uk/gwas/home, with GWAS IDs ranging from GCST90277238 to GCST90277416. The database for osteonecrosis comes from the FinnGen study (r10.finngen. Fi) and includes 297 cases with 411,884 controls. |
|  | b) | Report summary statistics for phenotypic exposure(s), outcome(s), and other relevant variables (e.g. means, SDs, proportions) | The original contributions presented in the study are included in the article/[Supplementary Material](https://www.ncbi.nlm.nih.gov/pmc/articles/PMC10883046/#s12). |
|  | c) | If the data sources include meta-analyses of previous studies, provide the assessments of heterogeneity across these studies | we applied Cochran's Q statistic to our MR-IVW analyses and Rucker's Q statistic to our MR-Egger analyses to determine the presence of heterogeneity among the instrumental variables |
|  | d) | For two-sample MR:  i.  Provide justification of the similarity of the genetic variant-exposure associations between the exposure and outcome samples  ii.  Provide information on the number of individuals who overlap between the exposure and outcome studies | The data utilized in this study were sourced exclusively from European population samples, which were drawn from independent GWAS databases. This approach was chosen to minimize overlap and reduce potential bias. |
| 11 | **Main results** |  |  |
|  | a) | Report the associations between genetic variant and exposure, and between genetic variant and outcome, preferably on an interpretable scale | The results are reported in the form of p-values along with OR (95% CI), and p-values along with beta (95% CI). |
|  | b) | Report MR estimates of the relationship between exposure and outcome, and the measures of uncertainty from the MR analysis, on an interpretable scale, such as odds ratio or relative risk per SD difference | Mendelian randomization estimation reports are detailed in Supplementary Table 4, Supplementary Table 5. |
|  | c) | If relevant, consider translating estimates of relative risk into absolute risk for a meaningful time period | The detailed please find in Supplementary Table 4, Supplementary Table 5. |
|  | d) | Consider plots to visualize results (e.g. forest plot, scatterplot of associations between genetic variants and outcome versus between genetic variants and exposure) | The results are visualized in Figure 1A, Figure 1B |
| 12 | **Assessment of assumptions** |  |  |
|  | a) | Report the assessment of the validity of the assumptions | Firstly, we selected the SNPs of 179 lipidomes (osteonecrosis) as instrumental variables, which have a strong association with exposure, allowing us to perform Mendelian randomization inferences, and the large F statistics indicate that these analyses will not be affected by weak instrument bias. Secondly, the selected SNPs were ensured to have no association with any confounding factors that could influence the relationship between exposure and outcome. Lastly, the SNPs were confirmed to only impact the outcome through exposure factors. |
|  | b) | Report any additional statistics (e.g., assessments of heterogeneity across genetic variants, such as *I^2^*, Q statistic or E-value) | The Cochran's Q statistic to our MR-IVW analyses and Rucker's Q statistic did not detect any heterogeneity and MR-Egger intercept test did not show any directional pleiotropy. |
| 13 | **Sensitivity analyses and additional analyses** |  |  |
|  | a) | Report any sensitivity analyses to assess the robustness of the main results to violations of the assumptions | Additional analysis methods can be found in Supplementary Table 4, Supplementary Table 5. |
|  | b) | Report results from other sensitivity analyses or additional analyses | Leave-one-out and funnel plot analysis method are detailed in Supplementary Figure 1, 2, 3, 4 |
|  | c) | Report any assessment of direction of causal relationship (e.g., bidirectional MR) | We applied a bidirectional Mendelian randomization analysis. First, a Mendelian randomization analysis was conducted to assess the causal relationship between 179 lipid traits and osteonecrosis. Subsequently, a reverse Mendelian randomization analysis was performed to evaluate the causal relationship between osteonecrosis and lipid traits. |
|  | d) | When relevant, report and compare with estimates from non-MR analyses | In this study, protein network and transcriptomic analysis were utilized for result validation. |
|  | e) | Consider additional plots to visualize results (e.g., leave-one-out analyses) | Leave-one-out and funnel plot analysis method are detailed in Supplementary Figure 1, 2, 3, 4. |
|  | **DISCUSSION** |  |  |
| 14 | **Key results** | Summarize key results with reference to study objectives | Our study has established credible causal relationships between specific lipidome subtypes and osteonecrosis through Mendelian randomization, clarifying the intricate interplay between them. These findings provide novel insights into the pathogenesis of osteonecrosis and identify potential therapeutic targets. Future studies will further investigate the mechanisms by which different lipidome affect the progression of osteonecrosis and explore the possibility of targeted interventions. |
| 15 | **Limitations** | Discuss limitations of the study, taking into account the validity of the IV assumptions, other sources of potential bias, and imprecision. Discuss both direction and magnitude of any potential bias and any efforts to address them | Firstly, to ensure an adequate sample of SNPs for analysis, we judiciously eased the thresholds for selecting instrumental variables, which might have marginally impacted the statistical power, rendering it relatively modest, although we minimized this effect by F-statistic screening. Secondly, Although the study boasted a substantial sample size, it is imperative to acknowledge that the participants were exclusively drawn from a European ancestry background, which could potentially constrain the universal applicability of our findings to diverse regions or ethnic populations. |
| 16 | **Interpretation** |  |  |
|  | a) | Meaning: Give a cautious overall interpretation of results in the context of their limitations and in comparison, with other studies | This comprehensive analysis not only highlights the intricate interplay between osteonecrosis and lipid metabolism but also underscores the potential of targeting these liposomes in future therapeutic strategies. |
|  | b) | Mechanism: Discuss underlying biological mechanisms that could drive a potential causal relationship between the investigated exposure and the outcome, and whether the gene-environment equivalence assumption is reasonable. Use causal language carefully, clarifying that IV estimates may provide causal effects only under certain assumptions | In the discussion section, we focus on the relevant studies concerning the relationship between lipid traits and osteonecrosis, as well as the existing mechanisms underlying the mutual effects of lipid traits on osteonecrosis. At the molecular level, evidence has also been provided to support the potential causal relationship between them. |
|  | c) | Clinical relevance: Discuss whether the results have clinical or public policy relevance, and to what extent they inform effect sizes of possible interventions | These insights provide a solid foundation for subsequent studies aimed at elucidating the underlying mechanisms driving these associations and developing targeted interventions to mitigate the impact of osteonecrosis on lipid profiles. |
| 17 | **Generalizability** | Discuss the generalizability of the study results (a) to other populations, (b) across other exposure periods/timings, and (c) across other levels of exposure | This study provides a comprehensive analysis of the causal relationship between lipidomes and osteonecrosis. However, it did not investigate the effects of varying exposure periods or levels. Furthermore, the study was limited to a European population, raising questions about its generalizability to other populations. |
|  | **OTHER INFORMATION** |  |  |
| 18 | **Funding** | Describe sources of funding and the role of funders in the present study and, if applicable, sources of funding for the databases and original study or studies on which the present study is based | No funding available. |
| 19 | **Data and data sharing** | Provide the data used to perform all analyses or report where and how the data can be accessed, and reference these sources in the article. Provide the statistical code needed to reproduce the results in the article, or report whether the code is publicly accessible and if so, where | The original contributions presented in the study are included in the article/[Supplementary Material](https://www.ncbi.nlm.nih.gov/pmc/articles/PMC10883046/#s12). Further inquiries can be directed to the corresponding author. |
| 20 | **Conflicts of Interest** | All authors should declare all potential conflicts of interest | The authors declare no conflict of interest. |

This checklist is copyrighted by the Equator Network under the Creative Commons Attribution 3.0 Unported (CC BY 3.0) license.

1. Skrivankova VW, Richmond RC, Woolf BAR, Yarmolinsky J, Davies NM, Swanson SA, et al. Strengthening the Reporting of Observational Studies in Epidemiology using Mendelian Randomization (STROBE-MR) Statement. JAMA. 2021;under review.

2. Skrivankova VW, Richmond RC, Woolf BAR, Davies NM, Swanson SA, VanderWeele TJ, et al. Strengthening the Reporting of Observational Studies in Epidemiology using Mendelian Randomisation (STROBE-MR): Explanation and Elaboration. BMJ. 2021;375:n2233.
