# Supplementary figures and images for: Lipid metabolism and osteonecrosis: unraveling causal mechanisms via multi-omics and mendelian randomization
Source: Front Physiol. 2025 Oct 23;16:1642153. doi: 10.3389/fphys.2025.1642153 (PMC12589827; doi:10.3389/fphys.2025.1642153)

Supplementary Figure 3 Leave-one-out analysis of causal effect of osteonecrosis on lipidomes

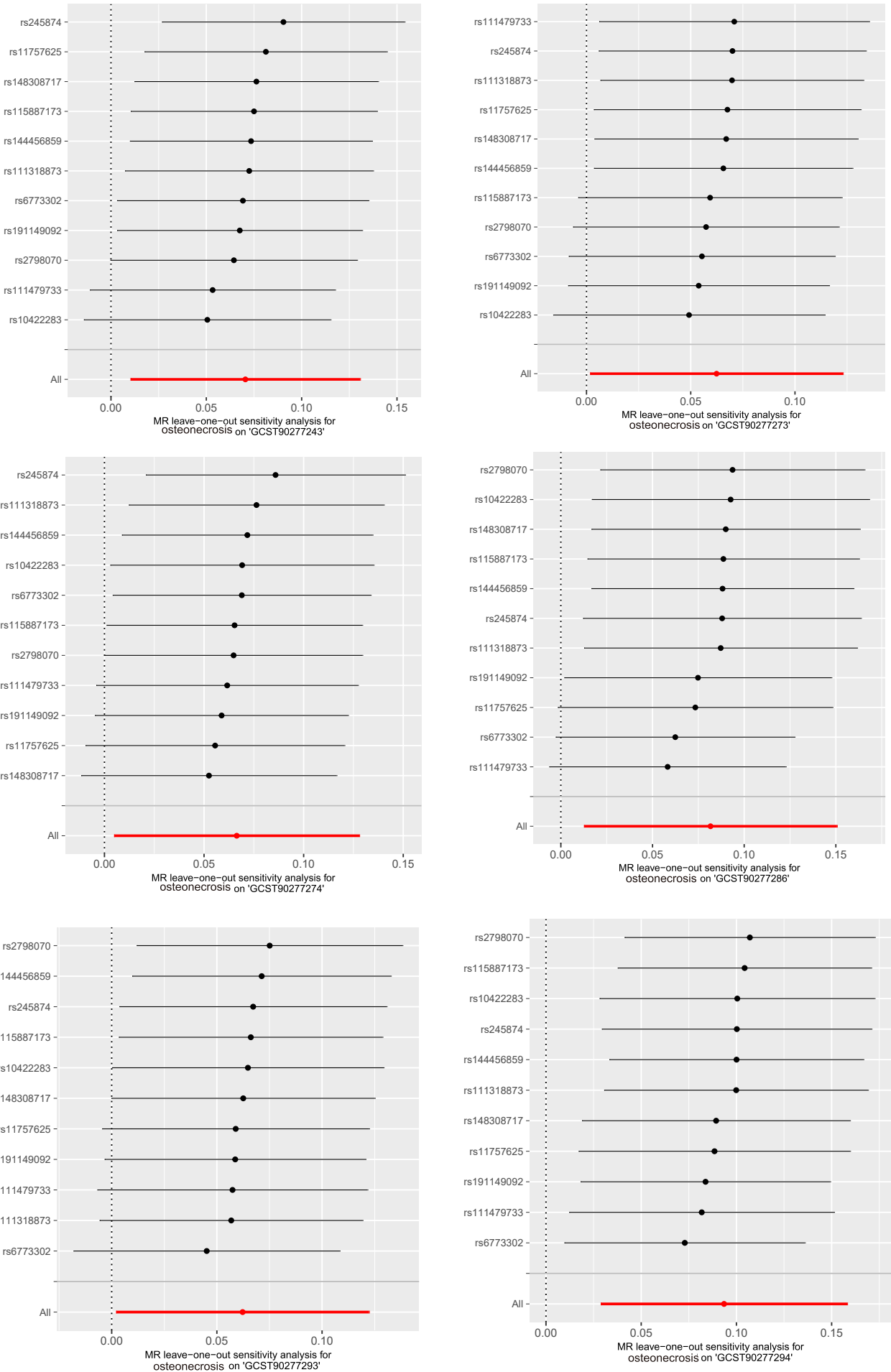

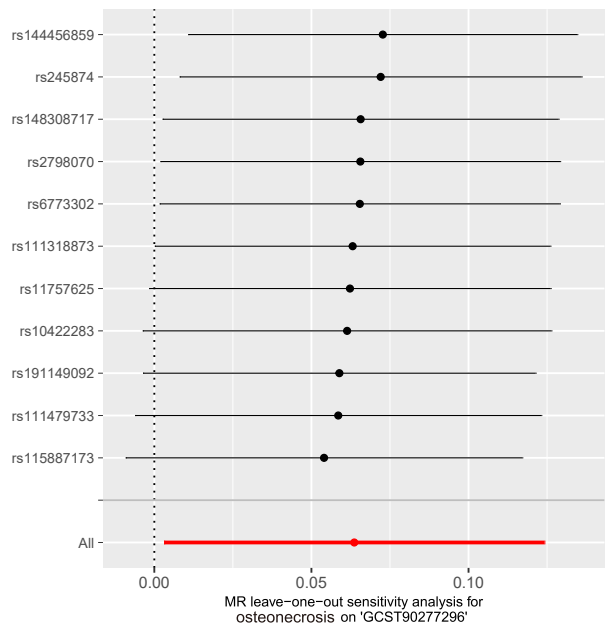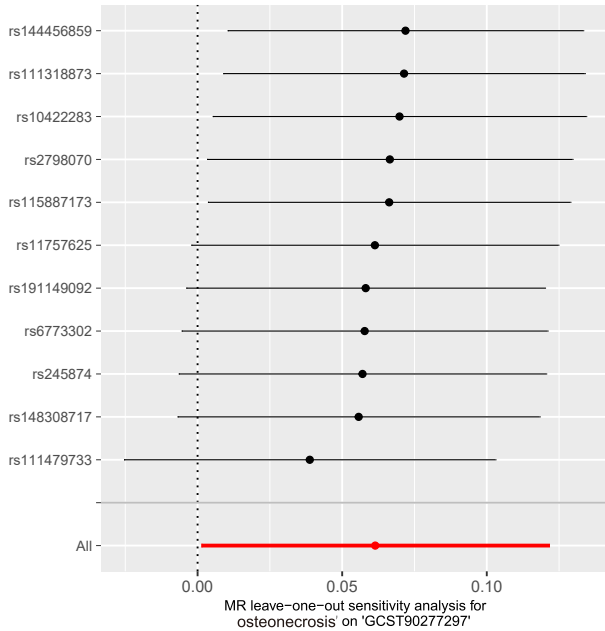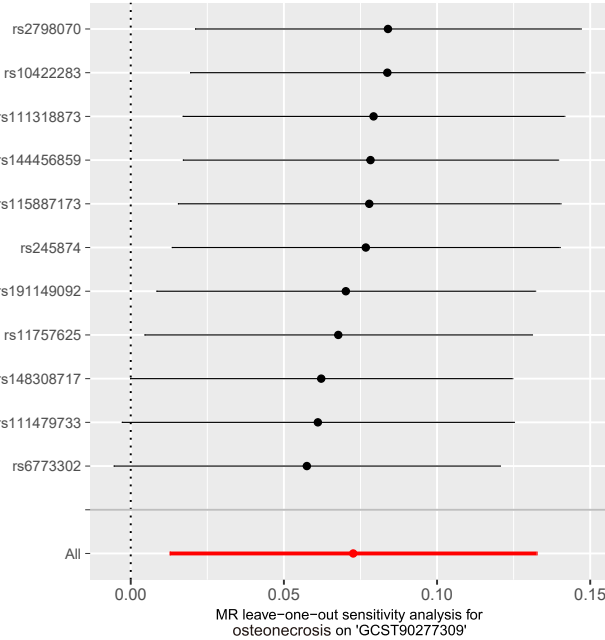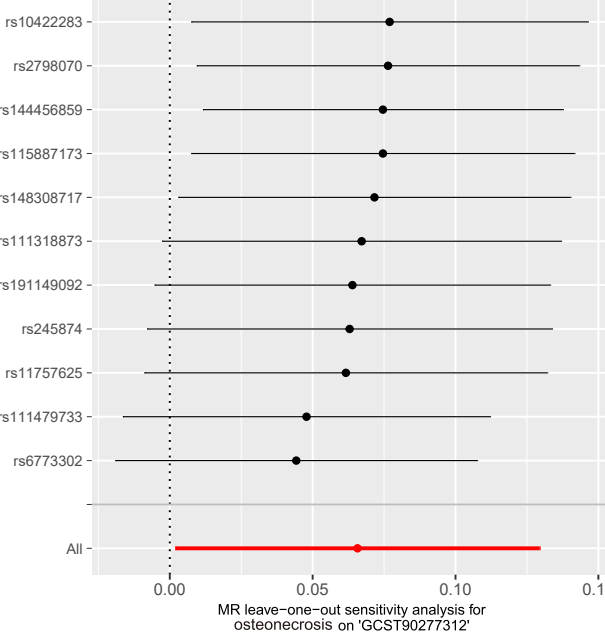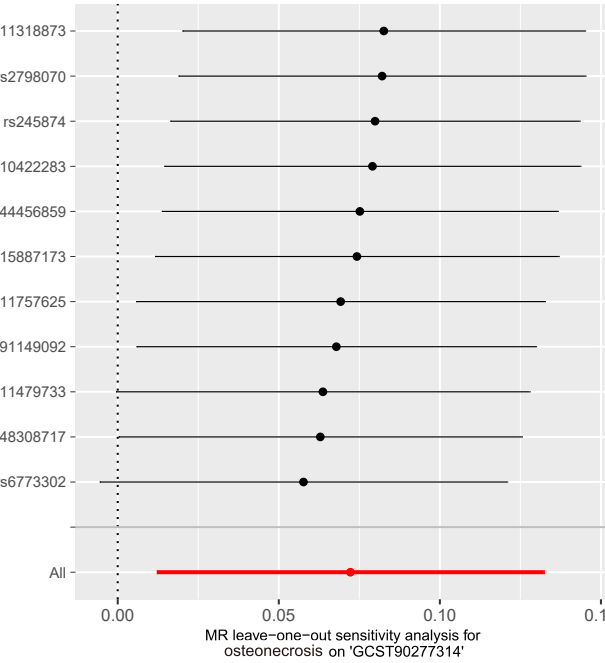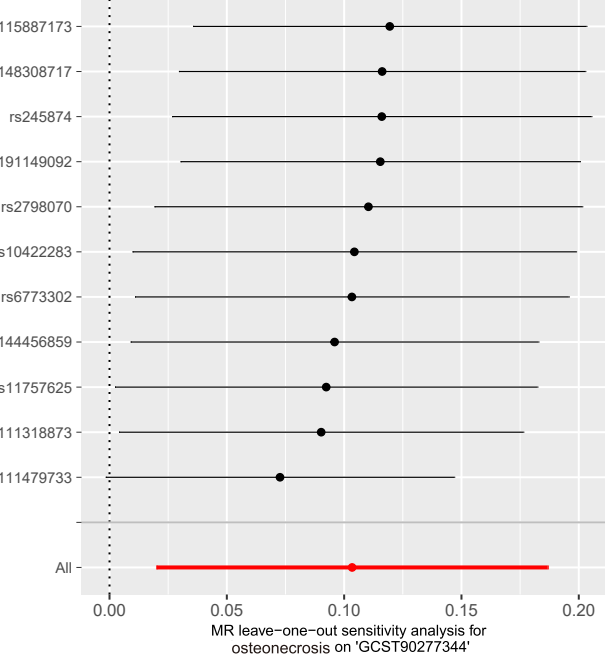

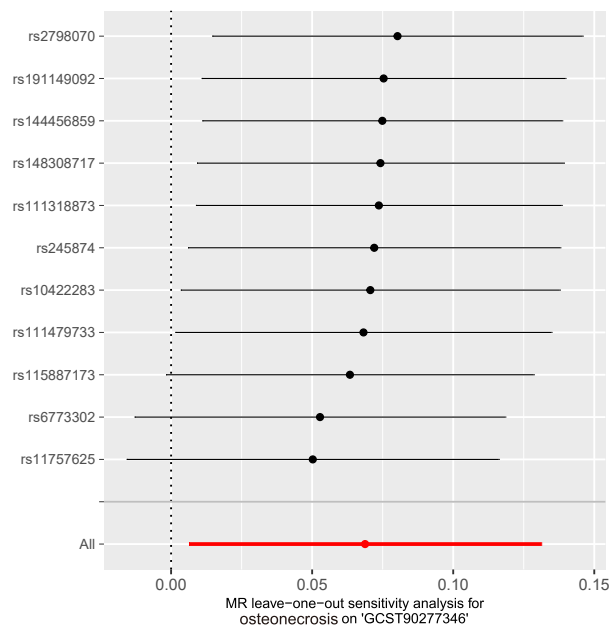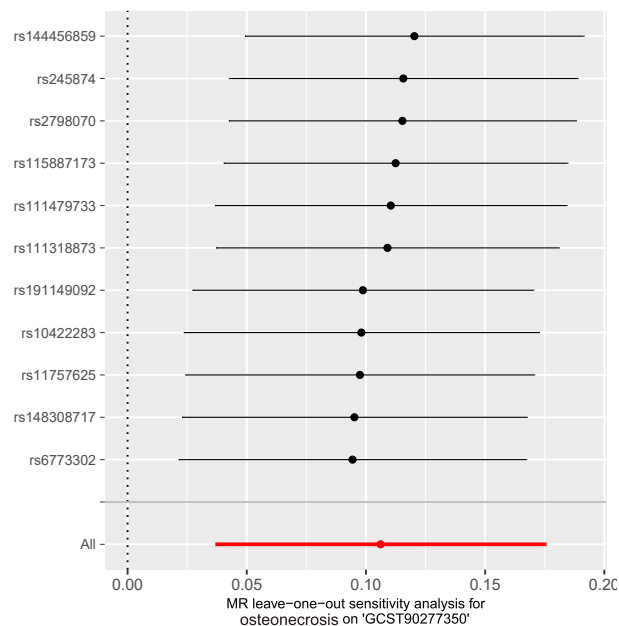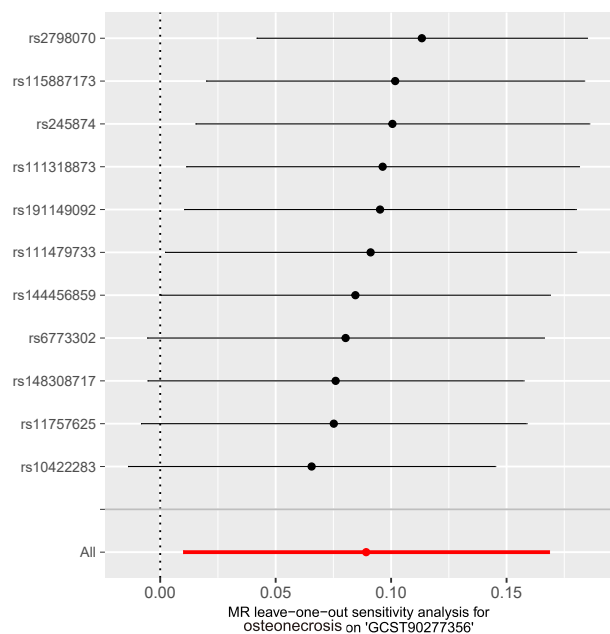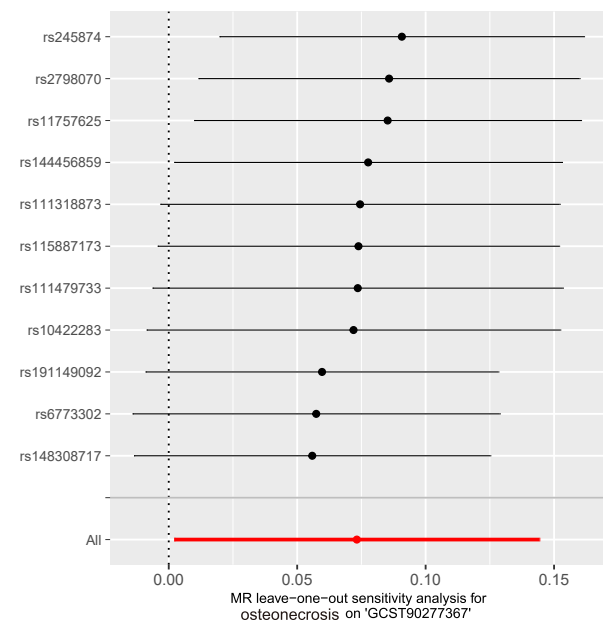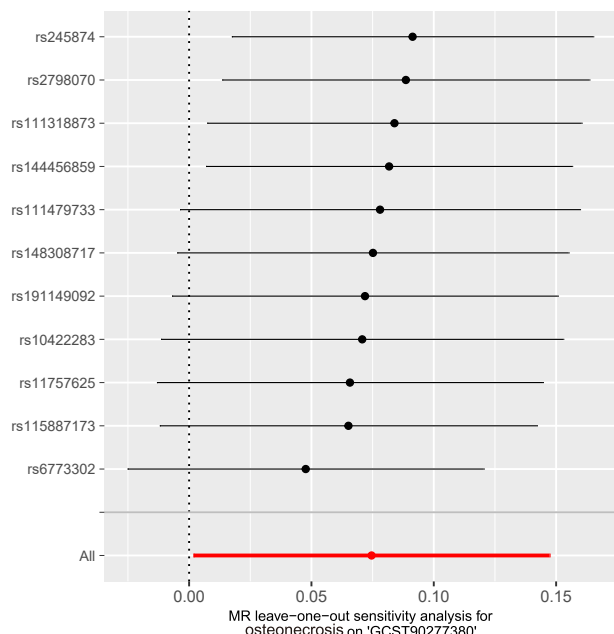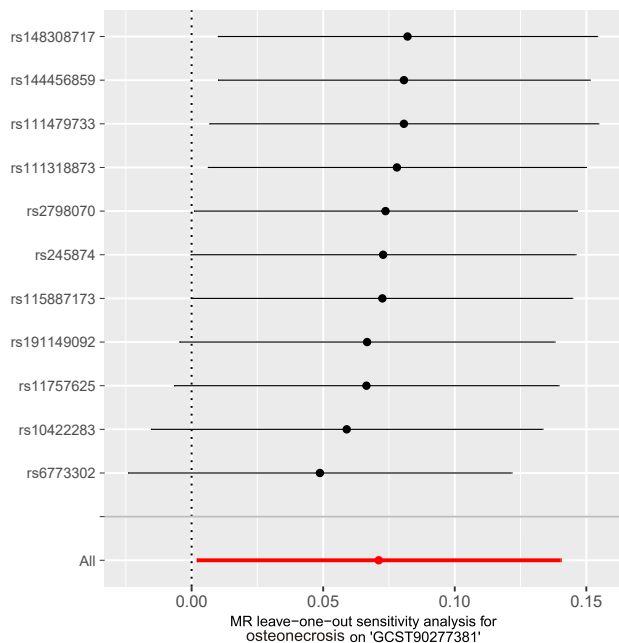

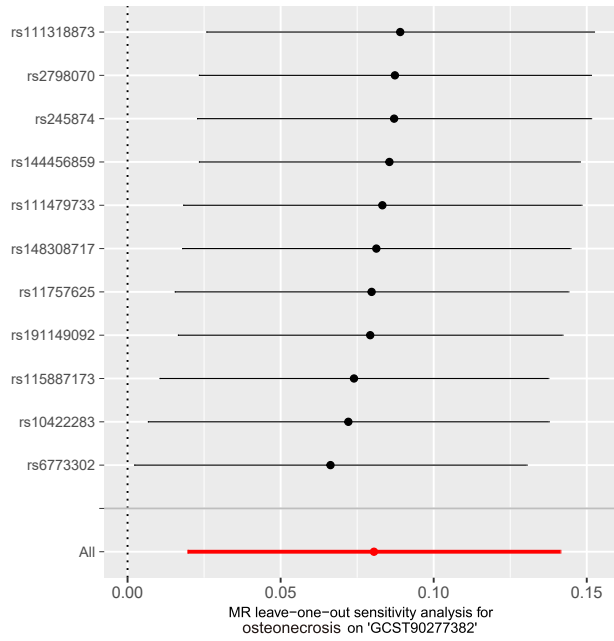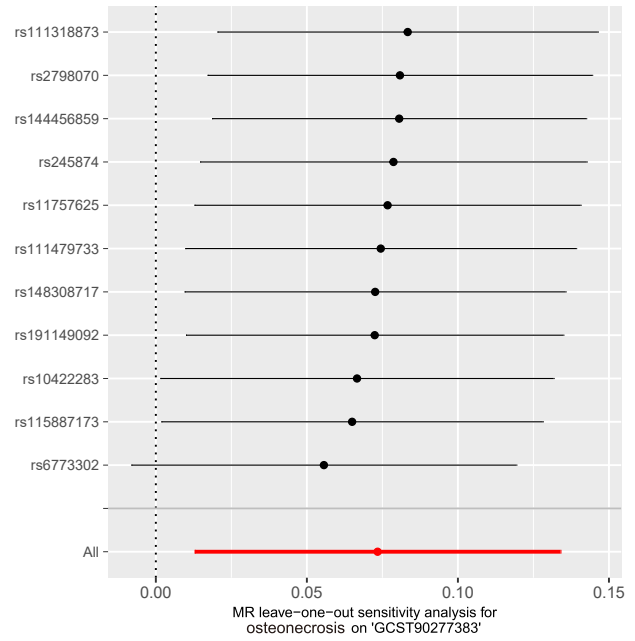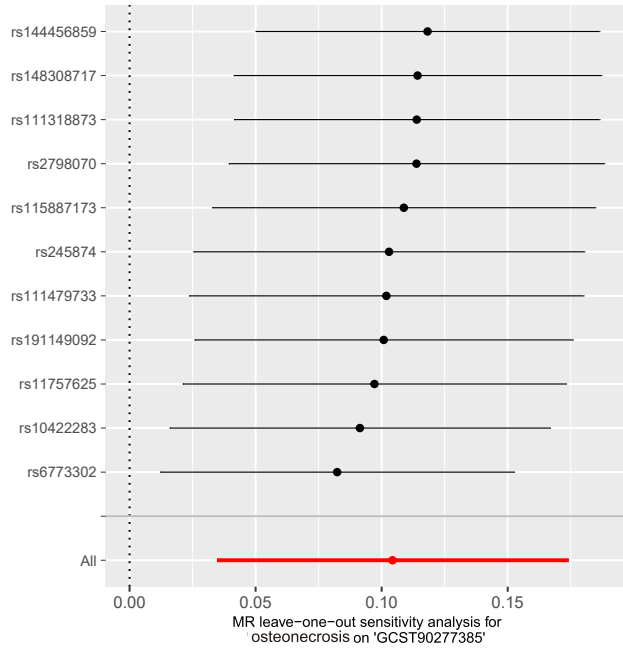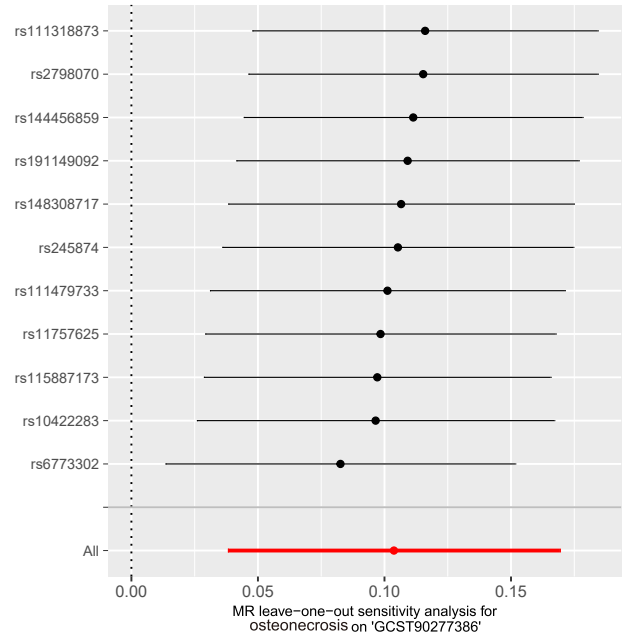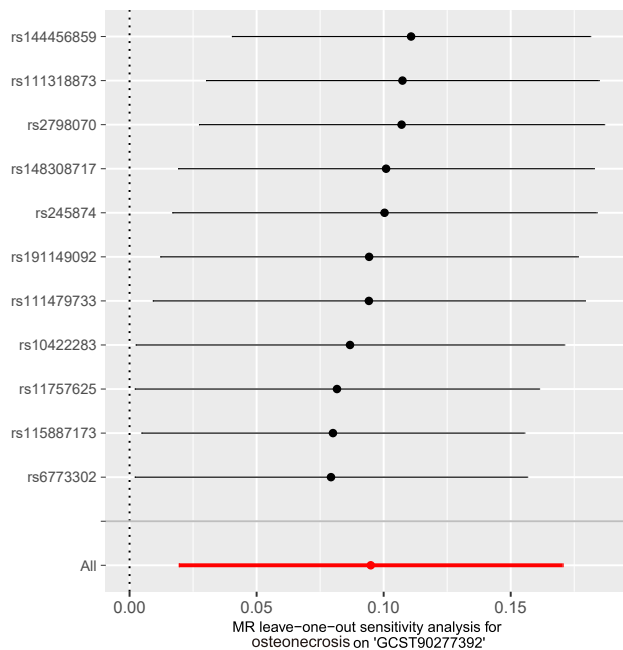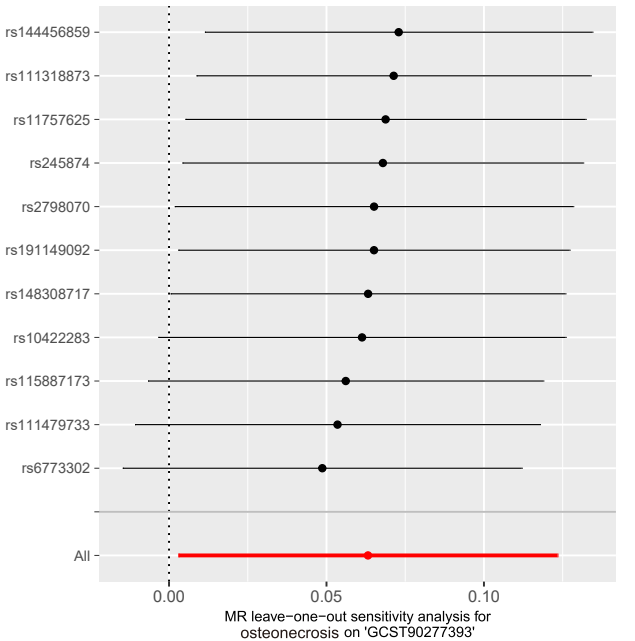

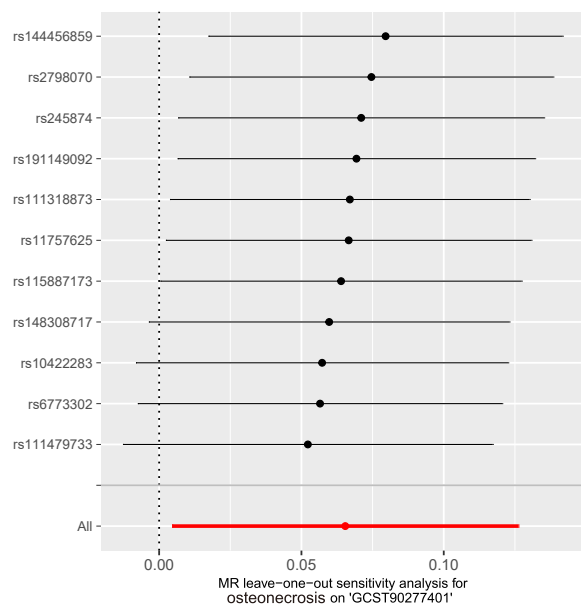

Supplement: Supplementary file 6 [file DataSheet3.pdf]

Supplementary Figure 1 Leave-one-out analysis of causal effect of lipidomes on osteonecrosis

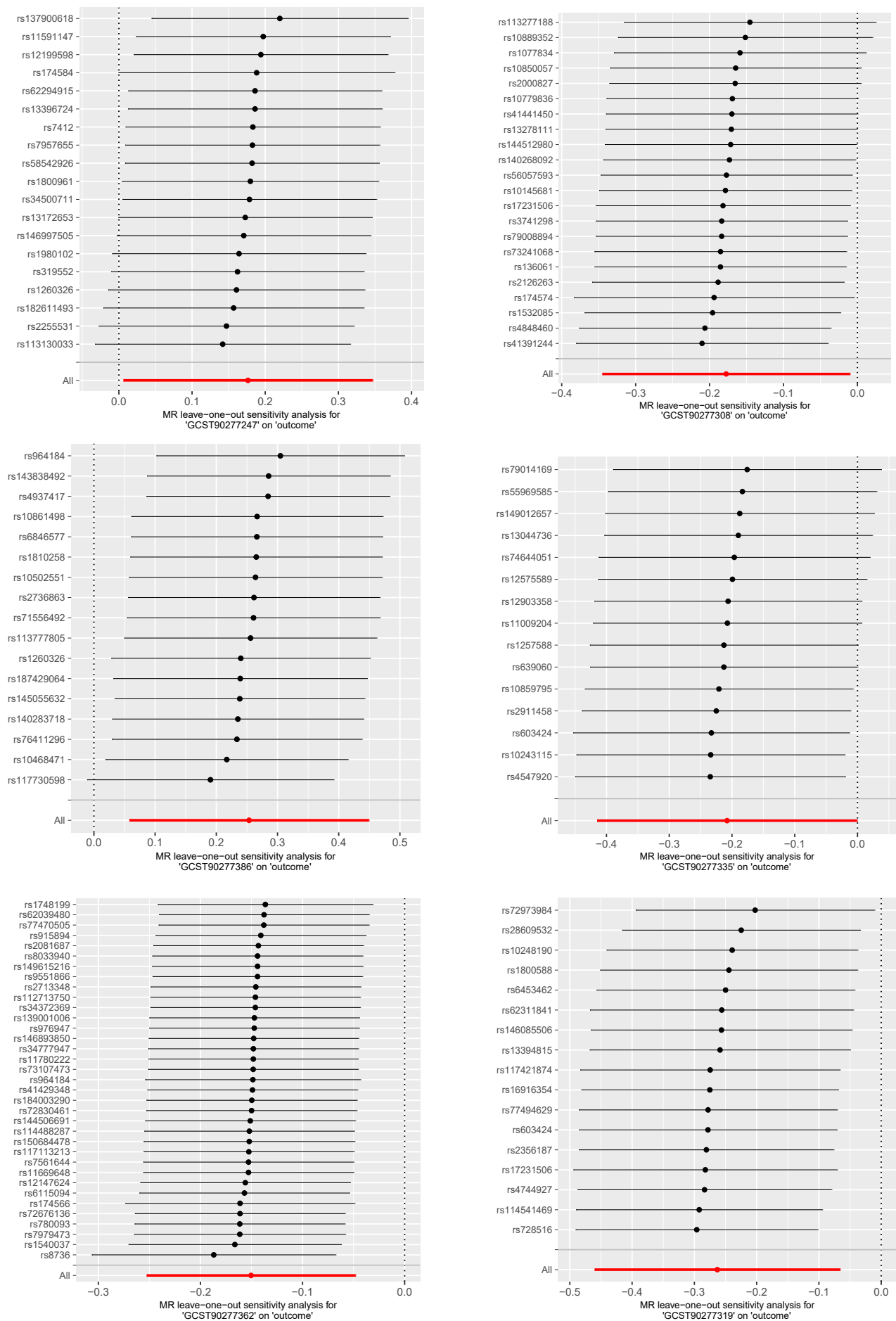

Supplement: Supplementary file 8 [file DataSheet1.pdf]
